# FACT Prevents the Accumulation of Free Histones Evicted from Transcribed Chromatin and a Subsequent Cell Cycle Delay in G1

**DOI:** 10.1371/journal.pgen.1000964

**Published:** 2010-05-20

**Authors:** Macarena Morillo-Huesca, Douglas Maya, Mari Cruz Muñoz-Centeno, Rakesh Kumar Singh, Vincent Oreal, Gajjalaiahvari Ugander Reddy, Dun Liang, Vincent Géli, Akash Gunjan, Sebastián Chávez

**Affiliations:** 1 Departamento de Genética, Universidad de Sevilla, Seville, Spain; 2 Department of Biomedical Sciences, College of Medicine, Florida State University, Tallahassee, Florida, United States of America; 3 Laboratoire d'Instabilité Génétique et Cancérogenèse, Institut de Biologie Struturale et Microbiologie, Centre National de la Recherche Scientifique, Marseille, France; Fred Hutchinson Cancer Research Center, United States of America

## Abstract

The FACT complex participates in chromatin assembly and disassembly during transcription elongation. The yeast mutants affected in the *SPT16* gene, which encodes one of the FACT subunits, alter the expression of G1 cyclins and exhibit defects in the G1/S transition. Here we show that the dysfunction of chromatin reassembly factors, like FACT or Spt6, down-regulates the expression of the gene encoding the cyclin that modulates the G1 length (*CLN3*) in START by specifically triggering the repression of its promoter. The G1 delay undergone by *spt16* mutants is not mediated by the DNA–damage checkpoint, although the mutation of *RAD53*, which is otherwise involved in histone degradation, enhances the cell-cycle defects of *spt16-197*. We reveal how FACT dysfunction triggers an accumulation of free histones evicted from transcribed chromatin. This accumulation is enhanced in a *rad53* background and leads to a delay in G1. Consistently, we show that the overexpression of histones in wild-type cells down-regulates *CLN3* in START and causes a delay in G1. Our work shows that chromatin reassembly factors are essential players in controlling the free histones potentially released from transcribed chromatin and describes a new cell cycle phenomenon that allows cells to respond to excess histones before starting DNA replication.

## Author Summary

Lengthy genomic DNA is packed in a highly organized nucleoprotein structure called chromatin, whose basic subunit is the nucleosome which is formed by DNA wrapped around an octamer of proteins called histones. Nucleosomes need to be disassembled to allow DNA transcription by RNA polymerases. An essential factor for the disassembly/reassembly process during DNA transcription is the FACT complex. We investigated a phenotype of yeast FACT mutants, a delay in a specific step of the cell cycle division process immediately prior to starting DNA replication. The dysfunction caused by the FACT mutation causes a downregulation of a gene, *CLN3*, which controls the length of that specific step of the cell cycle. FACT dysfunction also increases the level of the free histones released from chromatin during transcription, and the phenotype of the Spt16 mutant is enhanced by a second mutation affecting a protein that regulates DNA repair and excess histone degradation. Moreover, we show that the overexpression of histones causes a cell cycle delay before DNA replication in wild-type cells. Our results point out a so-far unknown connection between chromatin dynamics and the regulation of the cell cycle.

## Introduction

The FACT complex plays an important role by allowing RNA polymerase II (Pol II) to transcribe through chromatin (reviewed by [1], [2]), the only factor known to date that is able to stimulate Pol II-dependent transcription elongation through chromatin in a highly purified system [3], [4]. Yeast FACT is required *in vivo* to transcribe genes with highly positioned nucleosomes at the 5′ end of the transcribed region [5], and several lines of evidence of other organisms also support that FACT plays an important role in transcription elongation *in vivo* [6]–[10]. In spite of its role in elongation, several *in vivo* and *in vitro* approaches indicate an additional role of yFACT in establishing transcription initiation complexes by promoting TBP binding to core promoters in a TFIIA-dependent manner [11], [12]). Finally and in addition to its role in transcription, FACT also plays an important function during DNA replication [13]–[15].

In humans, the FACT complex is composed of two proteins, p140 and SSRP1, which are highly homologous to the essential yeast proteins Spt16/Cdc68/Ssf1 (hereafter referred to as Spt16) and Pob3, respectively [16]. *SPT16* had been previously identified as both a *CDC* gene [17], and also as a recessive suppressor of the deletion of *SWI4*, a transcription factor required for the high-level expression of the G1 cyclin genes, *CLN1* and *CLN2* [18]. Besides, Spt16 had also been described as a protein involved in transcription since several *spt16* alleles suppress the transcriptional effects of Ty insertions in yeast (Spt- phenotype) [19].

yFACT has been reported to interact physically or genetically with other factors related to histone modifications and chromatin remodeling, like the Paf complex, the ATP-dependent chromatin factor Chd1 and the NuA3 histone acetyltransferase complex [11], [20]–[22]. A reciprocal regulation of the FACT function by H2B ubiquitination has also been described [23]. In agreement with these findings, yFACT and the HMG-box protein Nhp6 have been shown to form a heterodimer capable of binding nucleosomes [24] and of reorganizing them *in vitro* [25], [26]. Both the yFACT subunits are able to bind H3/H4 tetramers and H2A/H2B dimers, sometimes in a functionally redundant manner [27], [28]. These interactions are thought to allow FACT to destabilize nucleosomes during transcription [26], [29].

Some *spt16* alleles are synthetically lethal with mutations affecting chromatin assembly [30]. Moreover, they lead to the activation of cryptic transcription initiation sites within coding regions, indicating that FACT, together with other factors like Spt6, also plays a role in maintaining the integrity of the chromatin structure during transcription [9], [31]–[34].

Several *spt16* mutants show defects while progressing through START, the main regulatory event in the G1 phase of the cell cycle [17], [35]. At a non-permissive temperature, the G1 phenotype of these *spt16* mutants has been accounted for by the drastic reduction in the expression of *CLN1*, *CLN2* and *CLN3*, the genes encoding the three G1 cyclins [35], [36]. *CLN1* and *CLN2* are able to self-regulate their expression by a positive feed-back mechanism [37], but the regulation of G1 length requires the activation of the cyclin-dependent kinase Cdc28 (Cdk1) by Cln3 [38]–[41]. Cln3-associated Cdk1 binds SBF (Swi4-Swi6) to the *CLN1* and *CLN2* promoters where it phosphorylates the negative regulator of START, Whi5 [42]. This phosphorylation promotes its release from SBF and leads to the activation of the *CLN1* and *CLN2* promoters [43], [44]. SBF-dependent recruitment of FACT plays an important role in this activation, which promotes the G1/S transition [45]. Notably, the kinase activity of Cln1,2-Cdk1 triggers the degradation of the cyclin-dependent kinase inhibitor Sic1 which no longer inhibits the S phase-promoting complex Clb5,6-Cdc28 [46], [47].

Another key regulatory process during the G1/S transition is the induction of histone genes, which allows the coupling of bulk histone synthesis to ongoing DNA replication. In proliferating cells, the synthesis of the vast majority of histones occurs during the S-phase of the cell cycle. The tight cell cycle regulation of the histone genes results from their transcriptional repression in phases G1 and G2, their transcriptional activation just before the S-phase and the post-transcriptional regulation of their mRNAs. During the S-phase, histone genes can also respond to changes; for instance, the accumulation of histones in response to the genotoxic agents interfering with DNA replication induces their repression (reviewed by [48]).

In recent years, a novel mechanism in budding yeast preventing the accumulation of free histones and which is superimposed upon the regulation of histone gene transcription and mRNA stability has been described [49]. This mechanism involves the use of the DNA damage checkpoint protein kinase Rad53 as part of a surveillance process that not only monitors the accumulation of excess histones, but also induces their degradation. This degradation is controlled by phosphorylation and is carried out by the proteosome in an ubiquitylation-mediated manner [50]. Excess histones are thought to be generated at the end of the normal S-phase or in response to an abrupt decrease of DNA synthesis following DNA damage. This mechanism is dependent on neither the checkpoint kinases Mec1 and Tel1 nor other DNA-damage checkpoint proteins (reviewed in [51]).

Some aspects of the G1 phenotype of the *spt16* mutants remain unknown. It is not clear whether FACT plays a direct role in the regulation of *CLN3*. Alternatively, the decreased expression of *CLN3* might be a physiological response to FACT inactivation. We investigated this question and found that the inactivation of FACT down-regulates the *CLN3* promoter in START. This phenomenon coincides with the accumulation of free histones and is enhanced by the mutation of the free histones controller, *RAD53*. We also found that the forced entry of FACT-deficient cells into the S-phase lowers their viability. Finally, we discovered that the overexpression of histones in the wild-type cells decreases *CLN3* transcription in START and leads to a delay in G1. We propose that the accumulation of free histones triggers the down-regulation of *CLN3*, thereby contributing to control the excess histones before starting DNA replication. We further propose that the main potential source of free histones is transcribed chromatin and that chromatin reassembly factors play an essential protective role in this respect.

## Results

### FACT inactivation causes a down-regulation of the *CLN3* promoter in START

**Figure 1:**
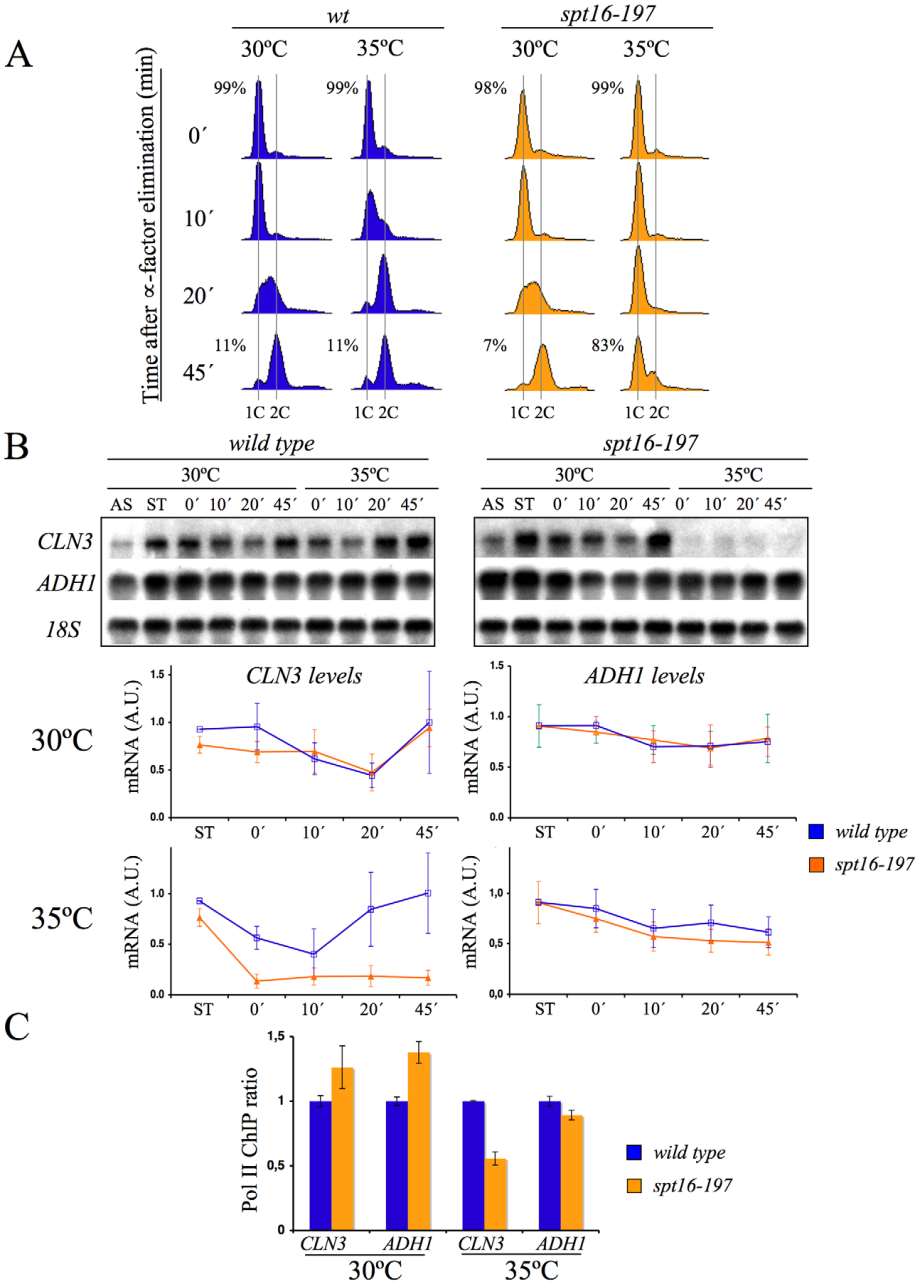
*CLN3* expression is down-regulated after Spt16 inactivation. Wild-type (FY120) and *spt16-197* (FY348) cells grown asynchronously (AS) were synchronized at START by treatment with alpha-factor for two hours at 30°C (ST), followed by one additional one hour at 30°C or 35°C in the presence of the mating pheromone. Cells were then released from the arrest at time 0 at either 30°C or 35°C by washing out the alpha-factor. Samples were taken at different time points to analyze the DNA content by flow cytometry and the proportion of unbudded cells by microscopy (A), and to quantify the mRNA levels of the indicated genes by Northern blot analysis (B). Transcripts levels were represented as arbitrary units (AU) after normalization with 18S rRNA. The results of a typical experiment and the quantification of three independent experiments are shown. (C) Relative RNApol II binding to the transcribed region of *CLN3* and *ADH1* in *spt16-197*. Cells were synchronized with alpha-factor for two hours at 30°C, followed by one additional one hour at 30°C or 35°C in the presence of the mating pheromone. The 8WG16 antibody, recognizing the CTD repeats of Rpb1, was used.

The arrest of yeast cells in G1 after Spt16 inactivation has been suggested to be a possible direct consequence of a very strict requirement of Spt16 for *CLN1*, *CLN2* and *CLN3* transcription [35]. FACT has been shown to participate directly in the activation of the *CLN1* and *CLN2* promoters after START [45]. In order to explore the effect of FACT inactivation on the previous step, we quantified the mRNA levels of *CLN3* in alpha factor-synchronized *spt16-197* cells at a non-permissive temperature (see Materials and Methods for the ranges of permissive and non-permissive temperatures ranges). We treated cells for two hours with the pheromone at 30°C, then for one a further hour at either 30°C or 35°C in the continuous presence of alpha-factor. Next we released the cells from the arrest and analyzed *CLN3* mRNA by Northern blotting (Figure 1). In agreement with previously published results based on asynchronous cells [35], the *CLN3* mRNA levels in the pheromone-treated *spt16-197* cells at 35°C were clearly lower than in the wild-type cells (see time 0 in Figure 1B). When cultures were released from alpha-factor, the wild-type cells progressed into the S-phase at any temperature, whereas *spt16-197* cells entered the S-phase only at 30°C, with most *spt16-197* cells remaining in G1 at the restrictive temperature (see times 10, 20 and 45 min in Figure 1A). The *CLN3* mRNA levels in the *spt16-197*cells released from alpha-factor at 37°C remained very low. In contrast, the mRNA levels of *ADH1*, a constitutive non-cycling gene, were only partially affected by Spt16 inactivation (Figure 1B), which is in agreement with the limited effect of *spt16-197* at restrictive temperatures in a broad set of non-cyclin genes [35], [36]. The occupancy of *CLN3* and *ADH1* by the RNA polymerase II (RNApol II) paralleled their mRNA levels. As Figure 1C depicts, the amount of RNApol II bound to the *CLN3* transcribed region at 35°C was significantly lower in *spt16-197* (52%) than in the wild type, whereas the variation of RNApol II binding to *ADH1* was slighter.

**Figure 2:**
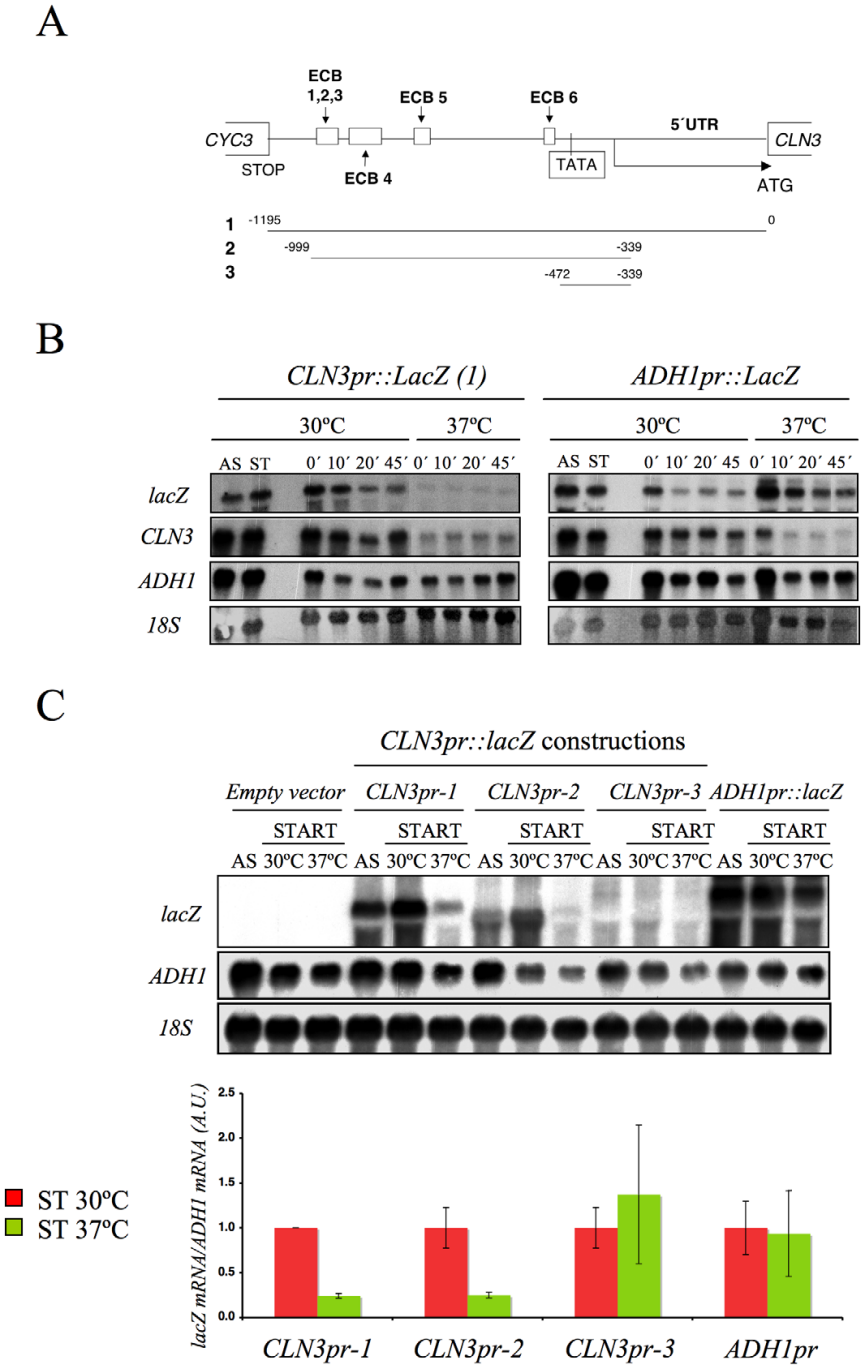
The *CLN3* promoter mediated the down-regulation of its mRNA levels after Spt16 inactivation. (A) Diagram of the plasmid-borne *lacZ* fusions, encoding *E. coli* ß-galactosidase, that were generated by introducing different fragments of the *CLN3* promoter region. Constructions were numbered 1 to 3. The positions of the early cell cycle boxes (ECB) and the TATA box are indicated. An *ADH1* promoter-*lacZ* fusion was used as a constitutively expressed control. (B) The mRNA levels of *CLN3*pr::*lacZ* and *ADH1*pr::*lacZ* fusion. The *spt16-197* (FY348) strain, transformed with the plasmids bearing *lacZ* fusion number 1 (*CLN3*pr::*lacZ*) or *ADH1*pr::*lacZ*, was grown until the mid-log phase (AS), and was then synchronized at START (ST) by alpha-factor treatment for 2h at 30°C, followed by one additional hour of incubation at 30°C or 37°C in the presence of the mating pheromone. Cells were then transferred to a fresh medium without alpha-factor (time 0h) to allow cells to progress at 30°C or 37°C. At the indicated times, samples were taken to analyze the transcript levels by Northern blot analysis with the indicated probes. The results of a typical experiment are shown. (C) The *spt16-197* (FY348) cells were transformed with the plasmids bearing the three *lacZ* fusions described in (A) or *ADH1*pr::*lacZ*. These transformants were grown until the mid-log phase (AS) and were synchronized with alpha-factor at 30°C (START), followed by an additional hour at 30°C or 37°C in the presence of the mating pheromone, as indicated. The transcript levels were quantified by Northern blot analysis. The results of a typical experiment and the quantification of three independent experiments are shown. The *CLN3* levels were normalized to *ADH1*. AU: arbitrary units.

The fact that the *CLN3* expression markedly reduced in response to Spt16 inactivation might be due to either a direct effect of FACT dysfunction on *CLN3* transcription or a yet unidentified signaling pathway targeting the *CLN3* promoter in response to FACT inactivation. In order to distinguish between these possibilities, we first analyzed the response of a reporter fused to the *CLN3* promoter upon Spt16 inactivation in alpha-factor-synchronized cells. To construct this fusion, we combined the entire intergenic region between the neighboring *CLN3* and *CYC3* coding regions and the coding region of *E. coli lacZ* (Figure 2A). We chose *lacZ* since we had previously showed that the transcription elongation of this reporter gene is not sensitive to FACT dysfunction [5]. As shown in Figure 2B, *lacZ* mRNA was hardly detectable when the synchronized *spt16-197* cells were incubated at the restrictive temperature, indicating that the *CLN3*pr::*lacZ* reporter (fusion 1) behaved similarly to the endogenous *CLN3* mRNA. In contrast, the mRNA levels of an *ADH1*pr::*lacZ* transcriptional fusion did not lower under the same conditions. This result indicates that the promoter region of *CLN3* mediates the drop in its mRNA levels after FACT inactivation.

**Figure 3:**
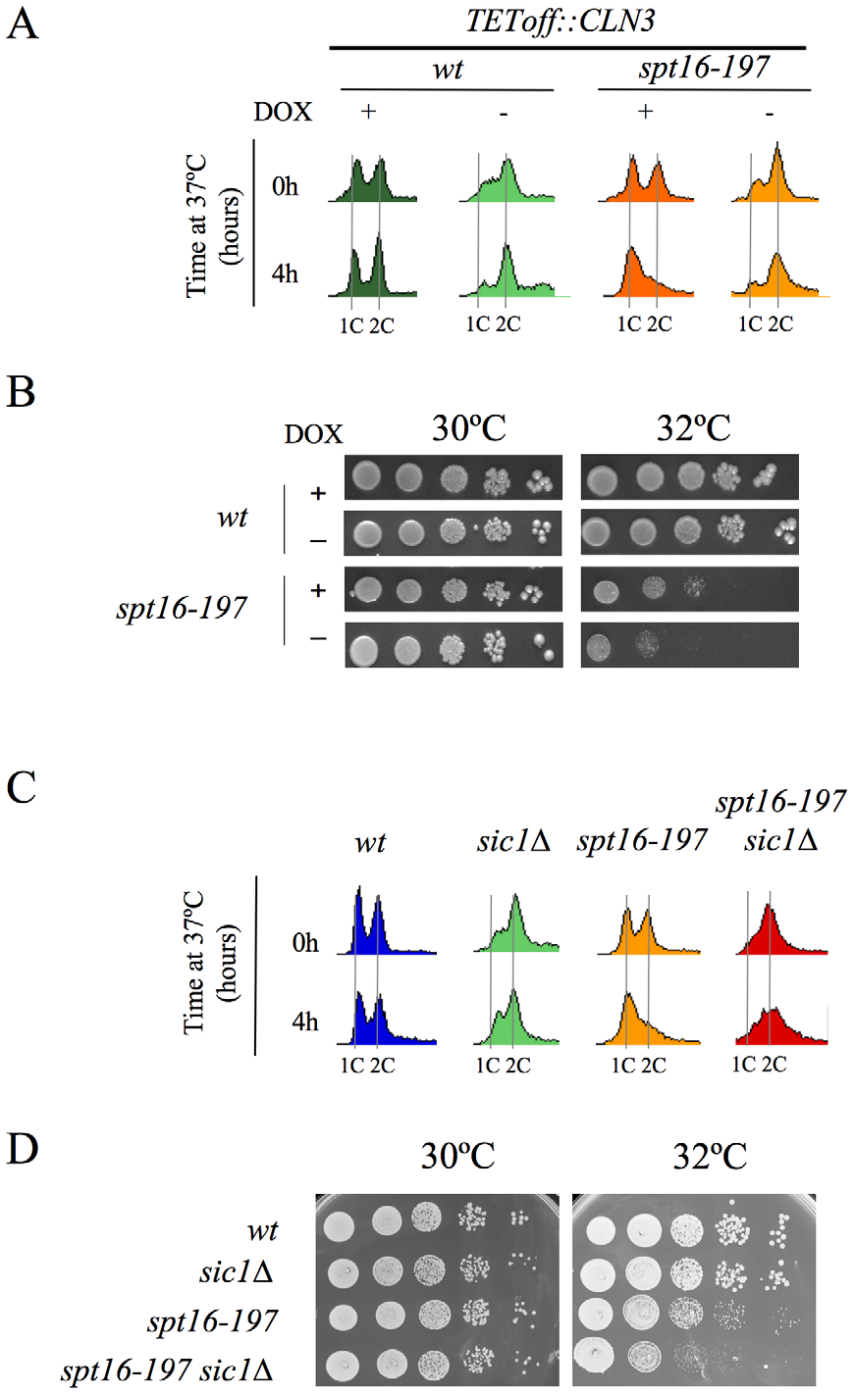
The forced entry of Spt16-deficient cells into the S-phase lowered their viability. (A) Wild-type (FY120) and *spt16-197* (FY348) cells, containing the centromeric *pTet_off_-CLN3-HA* plasmid, were grown in a synthetic medium (SC without uracil) at 30°C in the presence (+) or absence (−) of doxycycline (5µg/ml). Exponentially growing cells were then transferred to 37°C for four hours and analyzed by flow cytometry. (B) The wild-type and *spt16-197* cells containing the *pTet_off_-CLN3-HA* plasmid were grown until the mid-log phase in a synthetic medium (SC-URA) at 30°C in the presence (+) or absence (−) of doxycycline. Then, 10-fold serial dilutions were spotted onto the same media and incubated at the permissive (30°C) or semi-permissive (32°C) temperature. The first spot on the left corresponds to 5 µl of the undiluted culture. Pictures correspond to three days of incubation. No growth was detected on the forth spot of *spt16-197* minus doxycycline at 32°C, even after five days of incubation. (C) The wild-type (MMY18.10), *sic1Δ* (MMY18.12), *spt16-197* (MMY18.11) and *spt16-19 sic1Δ* (MMY18.9) cells growing exponentially in YPD at 30°C were shifted to 37°C for four hours. Cells were then analyzed by flow cytometry. (D) The exponentially growing cells from the strains mentioned in (C) were spotted onto YPD plates, as described in (B), and incubated at 30°C and 32°C as indicated. Pictures correspond to three days of incubation. No growth was detected on the fifth spot of the double mutant at 32°C, even after five days of incubation.

We further constructed additional *CLN3*pr::*lacZ* fusions containing different segments of the *CLN3-CYC3* intergenic region (Figure 2A). No difference in copy number among the reporter plasmids was detected by quantitative PCR (Figure S1). We measured the *lacZ* mRNA levels expressed by these fusions in the *spt16-197* cells synchronized in START. The expression patterns of the complete *CLN3*pr::*lacZ* (fusion 1) and *ADH1*pr::*lacZ* were in agreement with the experiments described above (Figure 2C). Fusion 2, which contains the entire intergenic region, except the *CLN3* 5′UTR and the *CYC3* termination region (−999, −339) (Figure 2A), retained sensitivity to Spt16 inactivation as it dropped as the *lacZ* mRNA levels did after Spt16 inactivation (Figure 2C). A significant influence of mRNA instability in this decrease was ruled out because two completely different mRNAs (*lacZ* and *CLN3*) responded similarly to FACT inactivation when they were driven by the same promoter (*CLN3*pr). In contrast, when *lacZ* mRNA was driven by the *ADH1* promoter, it did not show any significant variation in *spt16-197* at the restrictive temperature (Figure 2C). In short, *spt16-197* does not influence the expression of *CLN3* at a post-initiation level. However, Spt16 may also play a role in transcription initiation by facilitating TBP binding to the core promoters. We constructed *CLN3*pr::*lacZ* fusion number 3 (−472, −389), which contains the minimal core promoter of *CLN3* (Figure 2A). In spite of the weak expression of this fusion (five fold lower than fusion number 2 in asynchronous cultures), its mRNA level was not significantly influenced by Spt16 inactivation (Figure 2C). These results, in addition to the absence of FACT binding to the *CLN3* promoter during START (David Stillman, personal communication), indicate that the effect of Spt16 inactivation on the expression of *CLN3* in START is not due to the specific involvement of FACT in the transcription of this gene in this particular step of the cell cycle. Instead, our results suggest the existence of a control mechanism mediated by the promoter of *CLN3* which regulates the G1/S transition in response to FACT dysfunction. Such a mechanism might protect the cell from the deleterious effect of entering the S-phase under these conditions. We tested this hypothesis by forcing the entry of *spt16-197* cells into the S-phase. First, we went about this by overexpressing *CLN3* with a *Tet_off_::CLN3* construct (negatively regulated by doxycycline) which suppressed the accumulation of *spt16-197* cells in G1 at a restrictive temperature (Figure 3A). We observed a negative effect of the overexpression of *CLN3* on the viability of *spt16-197* cells at a semi-permissive temperature (Figure 3B). Similar results were obtained in those cells lacking Sic1, the inhibitor of the Cdc28-Clb complexes that negatively regulates entry into the S-phase (Figure 3C and 3D). These data reveal that the forced progression of *spt16-197* cells into the S-phase at semi-permissive temperatures is deleterious; therefore, the G1 delay triggered by the inactivation of FACT is cell-protective.

### The lethality associated with FACT dysfunction relates to the accumulation of the free histones evicted during transcription

**Figure 4:**
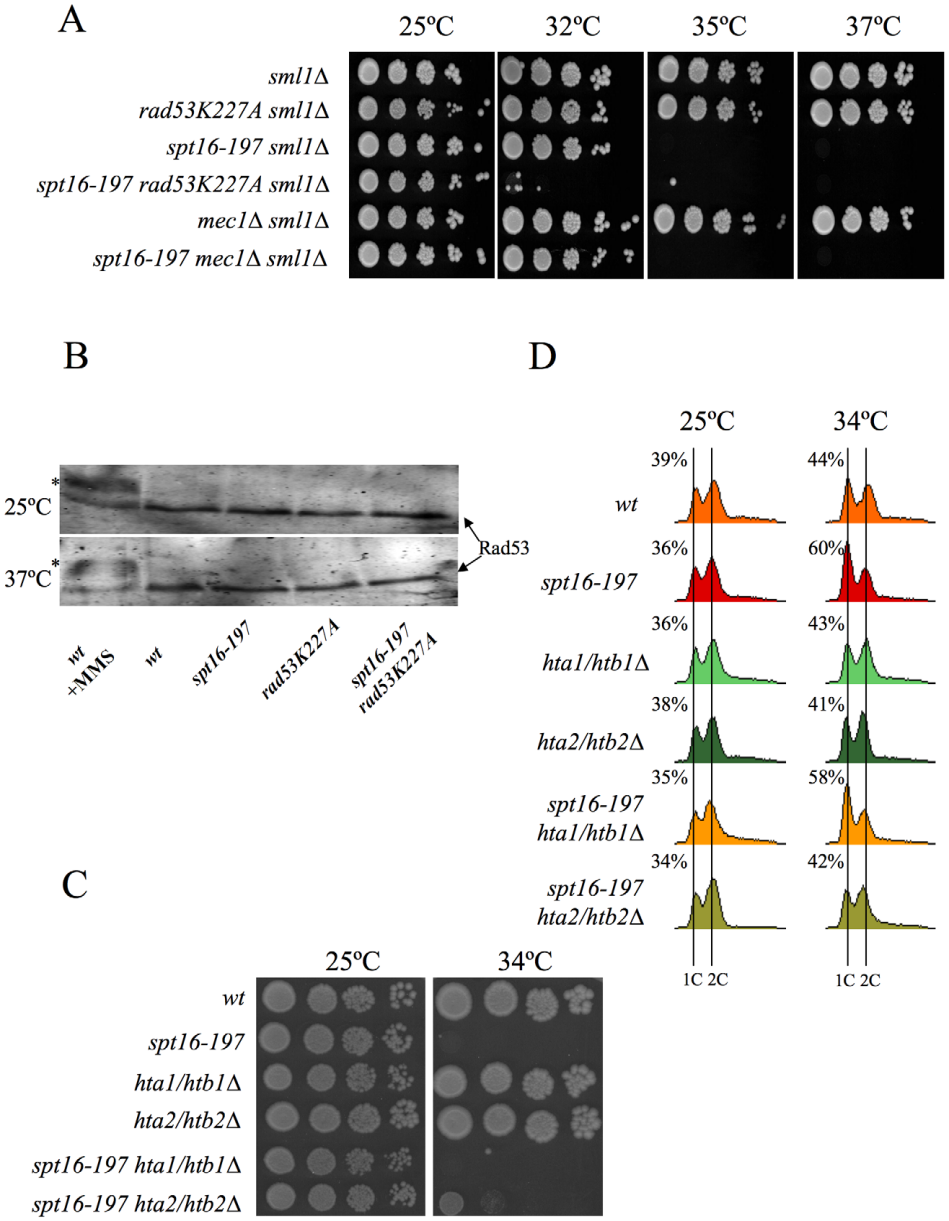
Genetic interactions connect FACT dysfunction to free histones. (A) *rad53K227A* enhances the thermosensitivity of *spt16-197*, irrespectively of the DNA damage checkpoint. Strains VO1-1, VO1-2, VO1-3, VO1-5, VO1-6, and VO1-8 were grown in YPD medium at 25°C. 10-fold serial dilutions were plated on YPD plates and incubated for 3 days at the indicated temperatures. (B) FACT dysfunction did not induce the hyperphosphorylation of Rad53p. The indicated strains (FY120, FY348, VO3, and VO7) were grown in YPD at either 25°C or 37°C for two hours. The TCA-treated protein extracts were analyzed by Western blot analysis with the goat anti-Rad53 polyclonal antibody. The hyperphosphorylation of Rad53 (shown by an *) was evidenced in the wild-type cells treated with 0.02% MMS. (C) *hta2/htb2Δ* partially suppressed *spt16-197*. Strains (FY120, FY348, FY710, DMY10, DMY11, and DMY12) were grown in YPD medium at 25°C. 10-fold serial dilutions of the indicated strain were plated on YPD plates and incubated for three days at the indicated temperatures. (D) Cells of strains FY120 (WT), FY348 (*spt16-197*), FY710 (*hta1Δ–htb1Δ*), DMY10 (*hta2Δ–htb2Δ*), DMY11 (*spt16-197 hta1Δ–htb1Δ*), and DMY12 (*spt16-197 hta2Δ–htb2Δ*), exponentially growing in YPD at 25°C, were shifted to 37°C for four hours or kept at 25°C. Samples were taken to analyze the DNA content by flow cytometry. Numbers indicate the proportion of G1 cells.

The association between impairment of transcription elongation and genome instability is well established [52]–[54]. Since FACT plays a role during elongation, we wondered whether the G1 delay induced by FACT inactivation could be due to the action of the canonical DNA-damage checkpoint. To test this hypothesis, we investigated the possible implication of Rad9, a component of the DNA-damage sensor machinery operating in G1 [55], [56]. We performed a FACS analysis with strains carrying the *spt16-197* and *rad9Δ* mutations. The profile of the *spt16-197 rad9Δ* double mutant after four hours at a restrictive temperature was almost identical to that of the single *spt16-197* mutant (Figure S2A), indicating that the G1 arrest produced by FACT inactivation is proficient in the absence of Rad9. Accordingly, no genetic interaction between *spt16-197* and *rad9Δ* was detected when we analyzed the thermosensitivity of the double mutant (Figure S2B). We further tested whether *spt16-197* exhibited any aggravation of its ts phenotype in the absence of Mec1, the key kinase of the DNA-damage checkpoint pathway [57]. As with *rad9Δ*, the deletion of *MEC1* in *spt16-197* did not affect its thermosensitive (*ts*) phenotype (Figure 4A).

It was intriguing to note that of all the DNA-damage checkpoint genes we tested, only the mutation of *RAD53*, which encodes another essential protein kinase for the DNA-damage checkpoint [58], led to a clear genetic interaction with *spt16-197*. Indeed the *rad53K227A* allele, which abolishes most Rad53 kinase activity, dramatically enhanced the thermosensitive phenotype of *spt16-197*. While the growth of the *spt16-197* and *rad53K227A* single mutants was virtually not affected at 32°C, the double *spt16-197 rad53K227A* mutant was unable to grow at this temperature (Figure 4A). Similar results were obtained with the complete deletion of *RAD53* (Figure S3). Unlike what happens following DNA damage, Rad53 was not hyperphosphorylated in the *spt16-197* mutant at a restrictive temperature (Figure 4B). Hence we conclude that Rad53 kinase activity is required to alleviate the deleterious effects of the *spt16-197* mutation, irrespectively of the role it plays in the DNA damage response.

As we mentioned in the Introduction, Rad53 is involved in the detection and subsequent degradation of excess histones without becoming phosphorylated and independently of its role in the DNA damage checkpoint [49]. Since FACT is involved in chromatin transactions during transcription, we hypothesized that the dysfunction of Spt16 might cause an increase in free histones, which would need to be targeted for degradation by Rad53. The mutations lowering the H2A–H2B dosage have been described to enhance the viability of the *spt16* mutants [30]. Accordingly, we observed how the deletion of *HTA2–HTB2*, one of the two loci encoding H2A and H2B, partially suppressed the ts phenotype of *spt16-197* at restrictive (Figure 4C) and semi-restrictive temperatures (Figure S4A). The *hta2Δhtb2Δ* deletion also suppressed the accumulation of *spt16-197* cells in G1 at a restrictive temperature (Figure 4D). In contrast, the deletion of the *HTA1–HTB1* locus caused no suppression in either the ts phenotype (Figure 4C and Figure S4A) or the G1 delay (Figure 4D). The *HTA1–HTB1* locus has been shown to be essential for viability and the *hta1Δhtb1Δ* strain we used (FY710) is only alive because of an extra-chromosomal copy of *HTA2–HTB2* [59]. We confirmed the presence of this extra-chromosomal copy of *HTA2–HTB2* not only in FY710, but in the isogenic *spt16-197 hta1/htb1Δ* double mutant (Figure S4B). The main difference between these two histone loci lies in their regulation. The expression of *HTA1–HTB1* is sensitive to the levels of histones, whereas *HTA2–HTB2* is not [60]. In order to properly compare the effect of the two loci on the ts phenotype of *spt16-197*, we engineered new strains containing all the viable combinations of the H2A/H2B-encoding loci. We found that the presence of two copies of the *HTA2–HTB2* (non responsive to free H2A and H2B) led to a more severe thermosensitivity than the presence of two copies of the *HTA1–HTB1* locus (repressible by free histones) (Figure S4A). Moreover the deletion of the regulatory sequence (NEG), which mediates the repression of *HTA1–HTB1* in response to histone levels, enhanced the ts phenotype of *spt16-197* (Figure S4A). Taken together, these results suggest that the accumulation of free H2A and H2B contributes to the lethality of *spt16-197* at high temperatures and that the accumulation of *spt16-197* cells in G1 responds to free histones.

**Figure 5:**
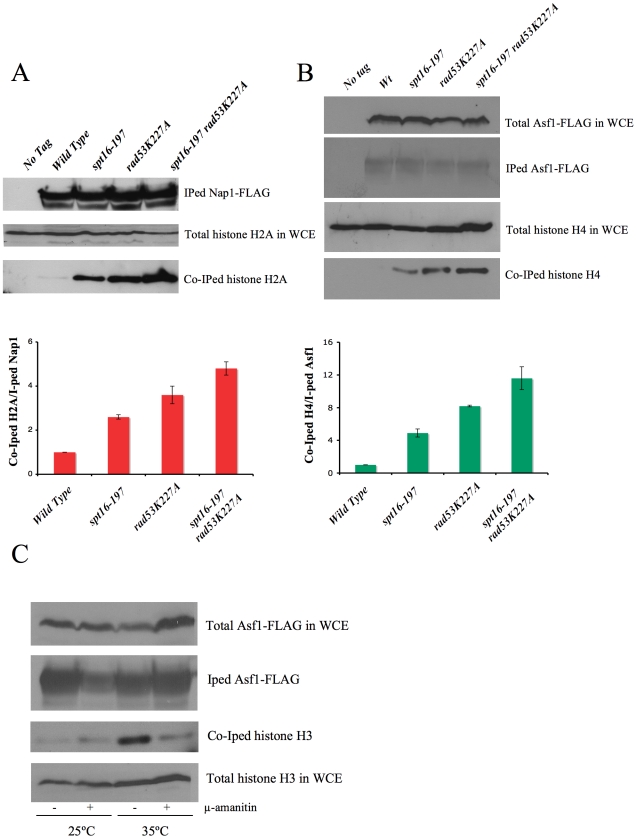
FACT dysfunction causes a transcription-dependent accumulation of free histones. (A) Histone H2A levels associated with chaperone Nap1. The indicated strains carrying Nap1-FLAG were grown exponentially at 25°C and then shifted for two hours to 37°C, prior to harvesting the cells for whole cell extract (WCE) preparation. Co-immunoprecipitation assays were carried out as described in the Materials and Methods. The levels of immunoprecipitated (IPed) Nap1 and co-immunoprecipitated (co-IPed) H2A are indicated. The histogram shows the relative accumulation of H2A on Nap1 compared to the wild-type cells (the data have been normalized to the amount of Nap1 actually IPed). The total levels of H2A in WCE are shown to demonstrate that roughly equal amounts of WCE were used for the immunoprecipitation reactions. (B) The histone H4 levels associated with Asf1 in the *spt16-197* cells. The indicated strains carrying FLAG-tagged Asf1 were grown in YPD media at 25°C, while the exponentially growing cells were harvested for WCE preparation. The co-immunoprecipitation assays for Asf1-H4 were carried out as indicated in the Materials and Methods. The total histone H4 and the total Asf1-FLAG levels in the WCE are shown to demonstrate that roughly equal amounts of WCE were used for the immunoprecipitation reactions. The histogram displays the relative accumulation of H4 on Asf1 if compared to the wild-type cells (the data have been normalized to the amount of Asf1 actually IPed). (C) The accumulation of the free histones in G1 upon FACT dysfunction is dependent on transcription. The *spt16-197* cells (FY348) carrying FLAG-tagged Asf1 were grown in YPD at 25°C, alpha factor-treated for two hours at 25°C, divided into four equal aliquots, and finally incubated with or without alpha-amanitin for two hours at 25°C or 35°C in the continued presence of the pheromone. Following this, cells were harvested and processed as described for (A). The Asf1-FLAG and histone H3 levels in WCE were shown to demonstrate that roughly equal amounts of WCE were used for the immunoprecipitation reactions.

In order to confirm this hypothesis, we analyzed the amount of non chromatin-associated histones in *spt16-197* in either the absence or presence of Rad53 kinase activity. The huge amount of histones present in the chromatin fraction makes it extremely difficult to quantify reproducibly free histones pools in a direct manner. Instead, we measured the amount of histones associated with soluble histone chaperones, a well-characterized procedure that allows a reproducible measurement of free histones [49], [50]. We performed co-immunoprecipitation assays using the H2A–H2B chaperone Nap1 (Figure 5A) and the H3–H4 histone chaperone Asf1 (Figure 5B) fused to the FLAG epitope. The *spt16-197* mutation did not produce a significant effect on the levels of Nap1-FLAG and Asf1-FLAG detected in the extracts (Figure S5A and S5B). We quantified the amount of H2A co-immunoprecipitated with Nap1 in exponentially growing cells after switching them to the non-permissive temperature. We saw a clear increase in the accumulation of free H2A in Nap1 in the *spt16* and *rad53* mutants compared to the wild-type cells, and an even higher level of co-immunoprecipitated H2A in the double mutant (Figure 5A). Next we performed a similar experiment with Asf1-FLAG. Wild-type cells exhibit very little H4 associated with Asf1 under normal growth conditions at 25°C (Figure 5B). We found that, even at this permissive temperature, the *spt16-197* mutation increased the amount of histones associated with Asf1 up to levels close to those shown by *rad53K227A* (Figure 5B). One again, the accumulation of free H4 in the double mutant exceeded the levels of the single mutants (Figure 5B). To test whether this increase of free histones in FACT-deficient cells was taking place in G1, independently of the histone synthesis that takes place during the S-phase, we incubated alpha factor-synchronized *spt16-197* cells for two hours at either 25°C or 35°C. As expected, the amount of free histones associated with Asf1 in the *spt16-197* mutant increased at the restrictive temperature. We also observed that inhibiting RNApol II transcription with alpha-amanitin prevented this increase (Figure 5C). Northern blot experiments showed no misregulated expression of the histone genes in *spt16-197* during START (Figure S5D). Taken together, these results are compatible with a scenario in which Pol II-dependent transcription in the absence of active FACT causes an accumulation of the evicted histones, which become toxic to the cell if not targeted for degradation by Rad53.

### Excess histones induce a G1 cell cycle delay

**Figure 6:**
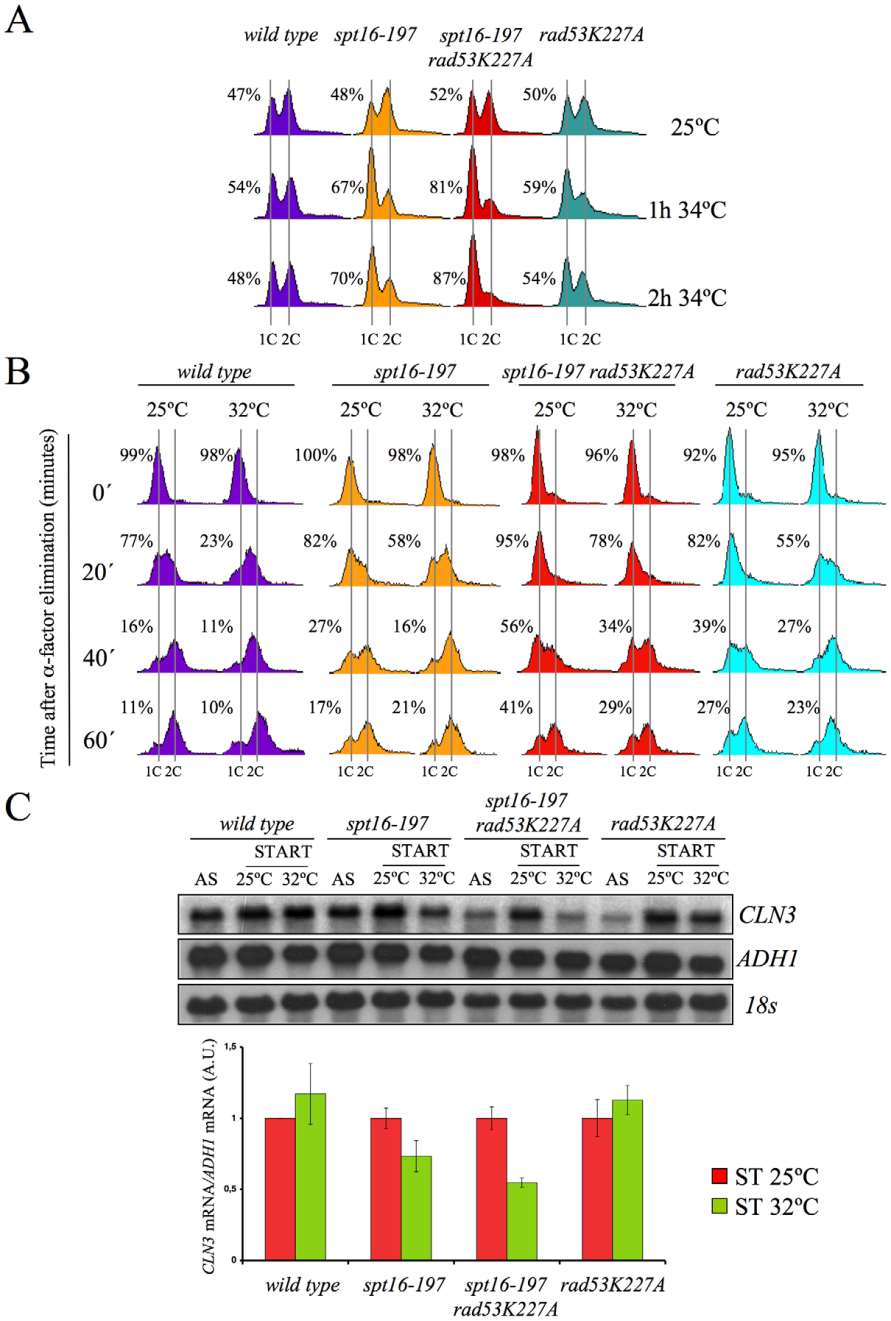
Excess histones induce a cell cycle delay in G1. (A) The *spt16-197* (FY348) and *spt16-197 rad53K227A* (VO7) cells growing exponentially in YPD at 25°C were shifted to 32°C at the indicated times. DNA content was analyzed by flow cytometry and the proportion of unbudded cells was quantified by microscopy. (B) The wild-type (FY120), *spt16-197* (FY348), *spt16-197 rad53K227A* (VO7) and *rad53-K227A* (V03) cells were synchronized at START by a treatment with alpha-factor for two hours at 25°C, followed by an additional one hour at 25°C or 32°C in the presence of mating pheromone. Cells were then released from the G1-arrest at time 0 at either 25°C or 32°C by washing out the alpha-factor. Samples were taken at the different time points to analyze the DNA content by flow cytometry and to measure the proportion of unbudded cells by microscopy. (C) Those samples from asynchronous cultures and from the time 0 of B were taken to analyze the *CLN3* mRNA levels by Northern blot. The results of a significant experiment and the average quantification of three independent experiments are shown. The *CLN3* mRNA levels were normalized to *ADH1*.

Our results pose an intriguing question about a possible link between the *CLN3*-dependent G1 delay and the presence of excess histones, both induced by FACT dysfunction. We addressed this question by testing whether the enhancement of the *spt16-197* thermosensitivity caused by *rad53K227A* correlated with the observed delay in G1. As Figure 6A depicts, an asynchronous culture of the double mutant exhibited a stronger and faster accumulation of cells in G1 compared to the single *spt16-197* mutant when they were shifted to a restrictive temperature. Moreover, the alpha-factor-synchronized *spt16-197 rad53K227A* cells also displayed a much slower entry into the S-phase than the single *spt16-197* or *rad53K227A* mutants when the mating pheromone was removed from the medium at a semi-permissive temperature. Even at 25°C, the double mutant displayed a slower exit from G1, although the double mutant exhibited a proportionally stronger defect in the G1-S progression at 32°C, as compared to the single mutants (Figure 6B).

In order to establish a correlation between the excess histones and the *CLN3* mRNA levels in START, we measured them under conditions in which the mutant's general transcriptional capacity is not significantly affected, but the level of free histones increases. To do this, we pre-synchronized the *spt16-197 rad53K227A* cells with alpha-factor and analyzed the *CLN3* mRNA levels one hour after switching them to 32°C in the continued presence of pheromone. The results show a more marked reduction of *CLN3* mRNA after one hour at 32°C in the double *spt16-197 rad53K227A* mutant than in the single *spt16-197* mutant (Figure 6C). Therefore, the absence of Rad53 kinase activity enhances the down-regulation of *CLN3* produced by the dysfunction of the *spt16-197* allele.

**Figure 7:**
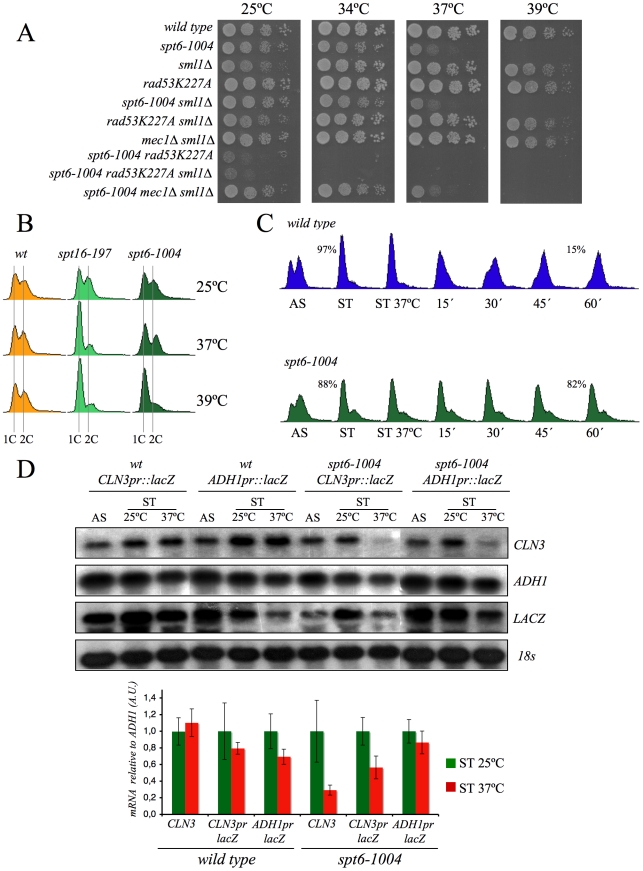
Spt6 dysfunction provokes G1/S defects and lowers cell viability in combination with *rad53K227A*. (A) Strains FY120, FY2180, VO1, VO3, DMY5, VO4, VO2, DMY6, DMY7, and DMY8 were grown in YPD at 25°C, spotted onto YPD plates and incubated for three days at the indicated temperature. (B) The wild-type (FY120), *spt16-197* (FY348), and *spt6-1004* (FY2180) cells exponentially growing in YPD at 25°C, were shifted for two hours at the indicated temperatures. Samples were then taken to analyze the DNA content by flow cytometry. (C) The wild-type (FY120) and spt6-1004 (FY2180) cells were synchronized at START by alpha-factor treatment for 2h at 25°C, followed by an additional one hour at 37°C in the presence of the mating pheromone. Cells were then released from the G1-arrest at time 0 at 37°C by washing out the alpha-factor. Samples were taken at the different time points to analyze the DNA content by flow cytometry and to measure the proportion of unbudded cells by microscopy. (D) The mRNA levels of *CLN3* and *CLN3*pr::*lacZ* in *spt6-1004*. The wild-type (FY120) and *spt6-1004* (FY2180) cells were transformed with the plasmids bearing the *CLN3*pr::*lacZ* fusion number 1 (see Figure 2A) or *ADH1*pr::*lacZ*. These transformants were grown until the mid-log phase (AS) and were alpha factor-synchronyzed at 25°C (START), followed by one additional hour at 37°C in the continued presence of pheromone. The RNA samples were taken to analyze the transcript levels. mRNAs were quantified by Northern blot analysis. The results of a typical experiment and the average quantification of three independent experiments are shown. The *CLN3* mRNA levels were normalized to *ADH1*, and the values of each strain at 37°C were represented in relation to 25°C.

If our hypothesis that transcriptionally evicted histones trigger the G1 delay of *spt16-197* is true, we should then expect the other mutants affected in chromatin reassembly to also exhibit similar cell cycle defects. In addition to FACT, another important factor that participates in chromatin reassembly during transcription is Spt6 [61]. We found that the viability of cells bearing the mutant allele *spt6-1004* clearly lowered in the absence of Rad53 kinase activity, but remained unchanged in the absence of Mec1 (Figure 7A). We also found that *spt6-1004* cells clearly accumulated in G1 when shifted to a restrictive temperature in both asynchronous and alpha factor-synchronized cells (Figure 7B and 7C). As in the *spt16-197* cells, the *CLN3* mRNA levels, or the *lacZ* mRNA levels when driven by the *CLN3* promoter, decreased when alpha factor-synchronized *spt6-1004* cells were shifted to a restrictive temperature (Figure 7D).

**Figure 8:**
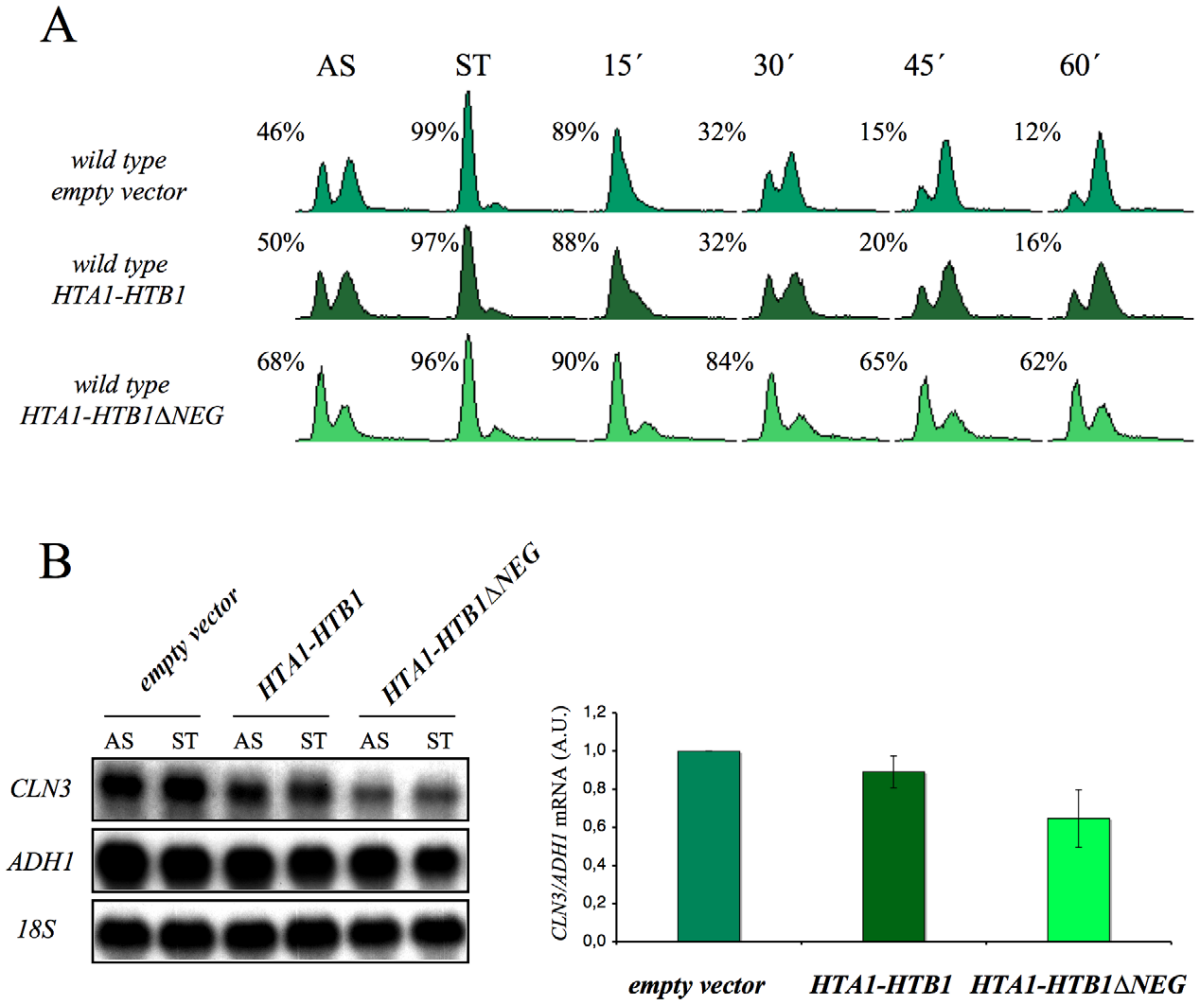
The overexpression of histones in the wild-type cells induces G1 delay. (A) Wild-type cells were transformed with pRS316 (empty vector) or with the analogous centromeric plasmids expressing either *HTA1–HTB1* or a mutant version of this locus lacking the sequence that mediates its transcriptional repression in response to the free histones (*HTA1–HTB1ΔNEG*). The transformants were grown exponentially in a selective medium (AS), synchronized at START by treatment with alpha-factor for two hours (ST) and released from the arrest by washing out the alpha-factor. Samples were then taken at the different time points to analyze the DNA content by flow cytometry and the proportion of unbudded cells by microscopy. (B) Aliquots from the synchronized cells used in (A) were taken to analyze the *CLN3* mRNA levels in START by Northern blot. The results of a typical experiment and the average quantification of three independent experiments are shown. The *CLN3* mRNA levels have been normalized to *ADH1*.

Given these results, we thought it would be interesting to determine whether the overexpression of histones in G1 induces a cell cycle delay in wild-type cells. We found that an additional copy of the *HTA1–HTB1* locus produced a very slight delay in cell cycle progression when alpha factor-synchronized cells were released from the pheromone. In contrast, a copy of the same locus lacking the regulatory sequence that mediates its repression in response to histone levels (ΔNEG) led to a clear accumulation of cells in G1 (asynchronous culture) and a more marked delay in the entry of synchronized cells into the S-phase (Figure 8A). Accordingly, we detected a more significant decrease of the *CLN3* mRNAs in START, in those cells bearing the deregulated *HTA1–HTB1* copy (ΔNEG) than in those transformed with the intact allele (Figure 8B). Since all the cells in this experiment were held in START by the presence of the mating pheromone, the drop in *CLN3* mRNA could not be an indirect consequence of free histones inhibiting cell cycle progression. We conclude, therefore, that excess histones downregulate *CLN3* in the wild-type cells during G1 and subsequently delay their progression through the G1/S transition.

## Discussion

The primary objective of our study was to understand the defects in the G1/S progression exhibited by the *spt16-197* mutant. The genetic and molecular evidence described in the Results section indicates that the dysfunction of Spt16 down-regulates the expression of *CLN3* in START by triggering a regulatory mechanism that specifically represses the *CLN3* promoter. Our results also reveal that the G1 delay undergone by the *spt16* mutant is not mediated by the DNA-damage checkpoint, although the *rad53K227A* mutation enhances both the thermosensitivity of *spt16-197* and its G1 phenotype. This result, in combination with the lack of phosphorylation of Rad53 after Spt16 inactivation, indicates that excess histones are involved in this phenomenon. We confirm that this hypothesis is true by showing that the Spt16 dysfunction produces an accumulation of free histones associated with histone chaperones in G1 (Figure 5C), and that excess histones induce a delay in the otherwise wild-type cells during the G1/S transition concomitantly with *CLN3* downregulation.

### Chromatin as a potential source of free histones

It is well known that the accumulation of non-nucleosomal histones in the cell is toxic [62] and that this toxicity is normally avoided by regulating the histone gene expression at both the transcriptional and posttranscriptional levels [60]. Obviously, these mechanisms are incapable of controlling excess histones when they originate by eviction from chromatin due to a dysfunction in chromatin reassembly during transcription. We demonstrate herein that Spt16 inactivation results in the accumulation of non-chromatin bound histones in G1. The only way for the cell to avoid the toxic effects of these excess histones is to degrade them in a process that is mediated by Rad53 [49]. Our results show that the absence of Rad53 kinase activity lowers the viability of the *spt16-197* mutant and suggest that one of the roles of the Rad53-dependent histone degradation mechanism is the elimination of those histones evicted by the transcriptional activity that are not reassembled into chromatin. Beyond the S-phase, transcribed chromatin is probably the main source of free histones in yeast cells, presumably due to minor imbalances between histone supply and demand during chromatin reassembly. The general similarities between histone trafficking during all the chromatin transactions suggests that DNA repair or DNA replication might also result in the excess of free histones when chromatin assembly is dysfunctional [63].

Our results suggest that FACT, in addition to avoiding initiation from cryptic promoters [9], [31], is a protective factor against the toxic risk represented by evicted histones. A recent publication reports how Spt16 promotes the redeposition of the original H3 and H4 histones evicted by elongating Pol II [34]. Our results agree with this conclusion since we have detected a clear accumulation of free H3 and H4 in Spt16-deficient cells. However, we have also noted an increase in the free H2A pool. This result and the genetic interactions between *spt16-197* and the H2A–H2B loci also indicate that Spt16 plays a role in preventing the accumulation of evicted H2A and H2B. In fact, a high H2A–H2B/H3–H4 gene ratio impairs the *spt16-11* growth, whereas a low H2A–H2B/H3–H4 gene ratio improves it [30], suggesting that the accumulation of free H2A–H2B caused by Spt16 dysfunction may be more relevant for the phenotypes observed.

It has been recently demonstrated that FACT promotes the transition between the canonical nucleosome configuration and a looser, more dynamic structure that involves changes in the interaction of the four core histones with the DNA [26]. According to this view, FACT would be essential for the maintenance of this altered configuration during transcription elongation by limiting the amount of the four histones joining the free pools. One prediction of this model is that those histone mutations which destabilize this alternative nucleosomal configuration would promote free histone accumulation. Some H4 mutations affecting H3–H4 tetramer/H2A–H2B dimer interactions show delayed G1/S transition and reduced *CLN3* expression levels [64]. Accordingly, we detected a negative synthetic interaction between one of these mutations (*hhf1-36*, bearing the H4-Y72G mutation) and *rad53K227A* (Figure S6).

The protective role against evicted histones is probably not an exclusive function of FACT, but is also a function of the other factors that cooperate during chromatin reassembly, like Spt6, for which we show some evidence. It is likely that Asf1, Nap1, the Hir complex or Nsr1 also protect against evicted histones. Asf1 has been seen to act as an intermediary in the parental histone disassembly/reassembly during replication as it associates with the MCM proteins at the replication fork [65]. An analogous situation might exist during transcription whereby Asf1 or the HIR complex, both of which have been shown to be capable of chromatin assembly outside the S-phase [66], [67], may act as an intermediary in the histone eviction and reassembly process in conjunction with FACT. Nap1 promotes nucleosome assembly by eliminating nonnucleosomal H2A- and H2B-DNA interactions and it prevents aberrant transcription by avoiding excessive H2A–H2B binding to DNA [68].

In this study we also show how the overexpression of H2A–H2B results in G1 delays in the wild-type cells. Similar effects, but to a lesser extent, were obtained by overproducing H3–H4 (data not shown). These results demonstrate that a histone-mediated G1 delay can be obtained in a background with no possible indirect effect mediated by either the role of FACT in the expression of the G1-S regulators, or the function of Rad53 in the control of early replication.

### *CLN3*-dependent G1 delay in response to free histones

Our results show that the G1-delay caused by Spt16 dysfunction correlates with a down-regulation of the transcriptional activity of the *CLN3* promoter. Cln3 shortage can only explain a transient G1-delay as cells can enter the S-phase after a prolonged G1 in the absence of Cln3. Therefore it is likely that the permanent G1 arrest shown by *spt16-197* cells at high temperatures (over 37°C) is caused by a combination of the *CLN3*-mediated transient delay and additional defects on the expression of other G1/S regulators caused by Spt16 inactivation. For instance, the MluI cell-cycle boxes (MCB)-mediated activation of *SWI4* and *SWI6* is also negatively affected by FACT dysfunction [18]. The *CLN1* and *CLN2* promoters are two other clear targets of Spt16 inactivation, since FACT binds them during G1 and participates in their activation after START [45]. However, the marked reduction in the *CLN3* expression following FACT inactivation cannot be caused by a direct involvement of FACT in the transcriptional activity of the *CLN3* promoter during G1 because it does not bind this promoter during this phase of the cell cycle (David Stillman, personal communication). We show herein that a *CLN3*pr::*lacZ* fusion lacking any sequence in common with *CLN3* mRNA is also responsive to the inactivation of Spt16 or Spt6 under conditions in which a constitutively expressed *ADH1*pr::*lacZ* is not. Furthermore, *rad53K227A* enhances the level of *CLN3* down-regulation caused by *spt16-197*. Finally, the overexpression of H2A–H2B in an otherwise wild-type strain decreases the *CLN3* expression. Taken together, these results support the existence of a mechanism which down-regulates the *CLN3* promoter during G1 in response to the accumulation of free histones caused by either defects in cotranscriptional chromatin reassembly or histone genes deregulation.

*CLN3* mediates several regulatory pathways by coupling the cell-cycle progression in G1 to physiological conditions, including nitrogen deprivation [69], daughter cell G1-delay [70], [71] and changes in glucose levels [72]. In the last case, the up-regulation of the *CLN3* promoter is due to the binding of the Azf1 transcriptional activator. Another transcription factor shown to regulate the *CLN3* promoter is the Mcm1-containing early cell-cycle box (ECB) binding complex [73]. Homeodomain repressors, Yox1 and Yhp1, restrict the ECB-dependent activation of the *CLN3* transcription to the M/G1 phase [74]. The deletion of *AZF1*, *YOX1* or *YHP1* does not alter the accumulation of cells in G1, as indicated by *spt16-197* at the restrictive temperature (data not shown). Moreover, the binding of Mcm1 to the *CLN3* promoter, measured by chromatin immunoprecipitation, was not affected by Spt16 inactivation (data not shown). We conclude that the transcriptional regulation of *CLN3* in response to the accumulation of free histones is not mediated by any of the known transcription factors operating on the *CLN3* promoter. It is even conceivable that the promoter itself acts as a sensor of free histone concentration and is repressed in response to the excess histones.

In mammalian cells, histone overexpression slows down entry into and progression through the S-phase [65]. Interestingly, depletion of human Spt16 leads to the repression of the H1, H2A and H2B genes [75], which could be the result of the accumulation of the free histones in human cells after FACT dysfunction. Given the analogy between the G1-S regulators in yeast (Cln3-SBF-Whi5-Rpd3) and mammals (CyclinD1-E2F-Rb-HDAC1) [42], [45], the functional link between the accumulation of free histones and the regulation of the G1-S transition may be evolutionarily conserved.

### Chromatin repair

The free histones evicted by the transcriptional activity of cells can potentially associate non-specifically with DNA via electrostatic interactions, and may give rise to aberrant chromatin structures which can be considered a form of chromatin damage. As with DNA damage, which enhances the risk of genome instability, excess free histones may have serious implications for the normal progression of DNA replication since the toxicity of free histones is maximal in the S-phase [49]. Consistently with this hypothesis, the experimental conditions under which the *spt16*-induced G1 delay is overcome (*CLN3* overexpression, *SIC1* deletion) involve an overall decrease in cell viability. The G1 delay should allow cells to reduce the free histone levels through the Rad53-mediated histone degradation pathway before entering the S-phase. The persistence of excess histones, as in the *spt16-197 rad53K227A* double mutant, would lead to severe replication dysfunctions. Accordingly, *chromatin repair* would be the combination of DNA repair, chromatin reassembly and excess histone degradation. In this sense, it is interesting to note that Rad53, which participates in both the DNA damage checkpoint and the excess histone degradation pathway, may act as a super-integrator of chromatin repair functions. This new concept could serve as a convenient framework to gain a better understanding of global genomic defects.

## Materials and Methods

For further details, see Text S1.

### Yeast strains and general procedures

All the yeast strains used in this study were derived from the S288C genetic background, unless otherwise indicated, and are listed in Table S1. In our background, temperatures over 33°C were restrictive for *spt16-197* growth, whereas temperatures below 31°C were permissive. All the experiments including *spt16-197* mutants were performed at several temperatures. For each experiment shown in the Results section, we chose the maximal restrictive temperature at which specific reproducible results were obtained. Standard procedures were followed for cell culturing, synchronization at START and flow cytometry [76], [77].

### Northern blot analyses

The Northern blot analyses were performed as previously described [5]. Six micrograms of total RNA prepared from yeast cells underwent electrophoresis on formaldehyde-agarose gels transferred to Hybond–N filters and UV crosslinked prior to hybridization at 65°C in 0.5M sodium phosphate buffer pH7 7% SDS with a [^32^P]dCTP-labeled DNA probe. Quantification of the mRNA levels was performed in a phosphorimager (FLA-3000, FujiFilm); the data are provided in arbitrary units. All the values were normalized in relation to the amount of 25S rDNA detected by hybridization with a ^32^P-oligolabeled 589 bp 25S rRNA internal fragment obtained by PCR and by using the 19-mer oligonucleotides TTGGAGAGGGCAACTTTGG and CAGGATCGGTCGATTGTGC. For the mRNA histone analysis, the whole coding regions of *HTA1* and *HHT1* were used as probes.

### Chromatin immunoprecipitation

Pol II ChIPs were performed as in [78], using the 8WG16 monoclonal antibody. Amplicons for Q-PCR quantification extended from +110 to +193 for *CLN3*, and from +6 to +95 for *ADH1*, in relation to the transcription start sites.

### Rad53p phosphorylation assay

Yeast cultures were grown at 25°C to OD = 1. Cultures were kept at 25°C, or shifted at 37°C and incubated for two hours. Protein extracts for the Western blot analyses were prepared from trichloroacetic acid (TCA)-treated yeast cells. Protein extracts were resolved on a 7.7% SDS-PAGE (35:0.2 acrylamide/bis-acrylamide). Immunoblots were done with the goat anti-Rad53 polyclonal antibody from Santa Cruz Biotechnology.

### Detection of non chromatin-bound “free” histones associated with the histone chaperones Nap1 and Asf1

For the determination of histones associated with Nap1 and Asf1 in Figure 5A and 5B, one-liter cultures of the indicated strains carrying Nap1-FLAG [79] or Asf1-FLAG were grown exponentially in YPD media at 25°C. Cells were then harvested as such at a density of 2.5×10E7 cells/ml for the Asf1-FLAG experiment shown in Figure 5B. For the Nap1-FLAG experiment shown in Figure 5A, cells were grown at 25°C until they reached a density of 1.5×10E7 cells/ml at which point they were switched to the restrictive temperature of 37°C for two hours prior to harvesting the cells at a density of 2.5×10E7 cells/ml. Whole cell extracts (WCEs) were prepared as previously described [49], and FLAG-tagged Nap1 (pRS316-Flag-yNap1) or Asf1-FLAG was immunoprecipitated using FLAG M2 agarose (Sigma). The immunoprecipitated material was resolved on precast 4–12% polyacrylamide gradient gels in MES buffer (BioRad), and were processed for Western blotting as previously described [49]. FLAG M2 antibodies (Sigma) were used to detect Nap1-FLAG and Asf1-FLAG, while histone H4 and H2B were detected using the previously described polyclonal antibodies [49]. Histone H2A was detected using an H2A antibody from Millipore (Cat. # 07-146).

The influence of transcription on free histone accumulation shown in Figure 5C was tested as follows. One liter of overnight culture of the *spt16-197* (FY348) cells carrying FLAG-tagged Asf1 was grown in YPD at 25°C. Once the cells had reached a density of 1.5×10E7 cells/ml, they were treated with alpha-factor for two hours at 25°C. Cells were treated with additional amounts of alpha-factor, divided into four equal aliquots, and were treated with or without 200µg/ml alpha-amanitin for 2 hours at 25°C or 35°C in the continued presence of alpha-factor. Afterward, cells were harvested and processed as described before, except histone H3, which was detected using a polyclonal antibody directed against the C-terminus of histone H3, as previously described [49]. The inhibition of Pol II by alpha-amanitin was controlled by monitoring the mRNA levels of *ACT1* in relation to ribosomal RNA levels (that are unaffected at the alpha-amanitin concentration used) using quantitative RT-PCR (Figure S5C).

## Supporting Information

Figure S1 Quantification of the relative copy number of the plasmids described in Figure 2. The indicated plasmids were detected by quantitative PCR as described in Text S1. The ratio between the amplicon localized in the Amp gene of the plasmid and another amplicon localized in the chromosomal *GAL1* genes is shown. 1.0 corresponds to the empty vector.
(0.18 MB PDF)

Figure S2 The G1 delay provoked by Spt16 inactivation was not prevented by the deletion of *RAD9*. (A) Wild-type (MMY20.4), *rad9Δ* (MMY20.1), *spt16-197* (MMY20.2) and *spt16-19 rad9Δ* (MMY20.3) cells growing exponentially in YPD at 30°C were shifted to 37°C for four hours. Cells were then analyzed by flow cytometry. (B) Wild-type and mutant cells exponentially growing in YPD at 30°C were spotted onto YPD plates and incubated at 30°C, 32°C and 33°C, as indicated.
(0.19 MB PDF)

Figure S3 *rad53Δ* enhances the thermosensitivity of *spt16-197* independently of the DNA damage checkpoint. Cells were grown in YPD medium at 25°C. 10-fold serial dilutions of the indicated strain were plated on YPD plates (or YPD+0.02% methyl methane sulfonate, MMS) and incubated for three days at the indicated temperatures.
(0.17 MB PDF)

Figure S4 *hta2/htb2Δ* partially suppresses the thermosensitivity of *spt16-197*. (A) Strains FY120, FY348, FY710, DMY10, DMY11, and DMY12 were transformed with pRS316 (empty vector), pRS316-HTA1-HTB1 or pRS316-HTA1-HTB1ΔNEG. Transformants were grown in SC-Ura medium at 25°C. 10-fold serial dilutions of the indicated strain were plated on SC-Ura plates and incubated for three days at the indicated temperatures. The chromosomal, extrachromosal or plasmidic copies of *HTA1–HTB1* and *HTA2–HTB2* presented in each transformant are indicated. Red squares indicate the results of those strains containing two copies of H2A/H2B-encoding loci. (B) An extrachromosomal amplification of the *HTA2–HTB2* locus was detected by PCR in FY710 (*hta1/htb1Δ*) and DMY11 (*spt16-197 hta1/htb1Δ*), following the protocol described in (Libuda and Winston, 2006).
(1.22 MB PDF)

Figure S5 *spt16-197* does not affect the levels of histone chaperones Nap1 and Asf1 or alter histone gene regulation. (A) Nap1 levels were unaffected in *spt16-197*. Nap1-FLAG was detected in WCEs of 4 independent generated wild type and spt16-197 strains by Western blotting using an anti-FLAG antibody. A non-specific band was used as the loading control. (B) Asf1 levels were unaffected in *spt16-197*. Asf1-FLAG was detected in WCE by Western blot with an anti-FLAG antibody. H2A was used as the loading control. (C) Alpha–amanitin inhibited RNA pol II transcription in alpha-factor-synchronized *spt16-197* cells. The mRNA levels of the constitutively expressed *ACT1* gene were measured by quantitative RT-PCR in the FY348 cells grown exponentially at 25°C and synchronized with alpha factor for four hours. The data was normalized to the levels of ribosomal RNA which is not affected by the concentration of alpha–amanitin used. (D) Repression of histones genes in G1 was not abolished by *spt16-197*. Wild-type (FY120) and *spt16-197* (FY348) cells grown asynchronously (AS) were synchronized at START (ST) by treatment with alpha-factor for two hours at 25°C (ST), followed by an additional one hour at 25°C or 35°C in the presence of the mating pheromone. Cells were then released from the arrest at time 0 at either 25°C or 35°C by washing out the alpha-factor. Samples were taken at the indicated time points to analyze mRNA levels by Northern blot. H2A indicate the signal corresponding to *HTA1* and *HTA2*, and H3 indicate the signal of *HHT1* and *HHT2*.
(0.24 MB PDF)

Figure S6 *hhf1-36* and *rad53K227A* exhibit a negative synthetic interaction. Strains MSY623, DMY15, MSY781, and DMY16 exponentially grown in YPD at 30°C were spotted onto YPD plates and incubated for three days at 30°C and 37°C, as indicated.
(0.16 MB PDF)

Table S1 The yeast strains used in this work.
(0.03 MB DOC)

Text S1 Supplementary material and methods and supplementary references.
(0.03 MB DOC)
