# Supplementary figures and images for: FACT Prevents the Accumulation of Free Histones Evicted from Transcribed Chromatin and a Subsequent Cell Cycle Delay in G1
Source: PLoS Genet. 2010 May 20;6(5):e1000964. doi: 10.1371/journal.pgen.1000964 (PMC2873916; doi:10.1371/journal.pgen.1000964)

**A**

RT-PCR of genomic DNA to compare levels of pRS416 to GAL1 gene in spt16-197 mutant

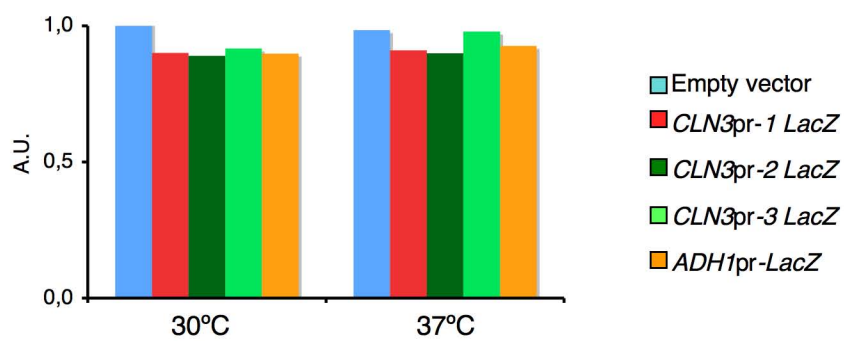

Figure S1

Supplement: Figure S1 — Quantification of the relative copy number of the plasmids described in Figure 2. The indicated plasmids were detected by quantitative PCR as described in Text S1. The ratio between the amplicon localized in the Amp gene of the plasmid and another amplicon localized in the chromosomal GAL1 genes is shown. 1.0 corresponds to the empty vector. (0.18 MB PDF) [file pgen.1000964.s001.pdf]

A

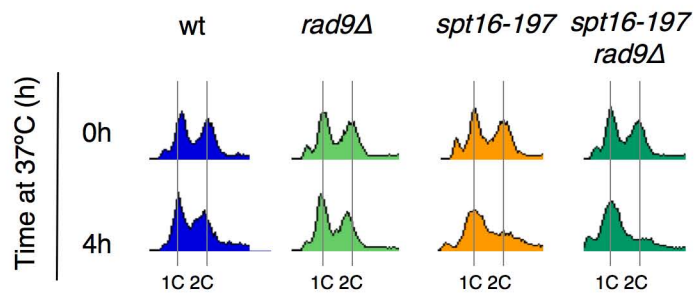

B

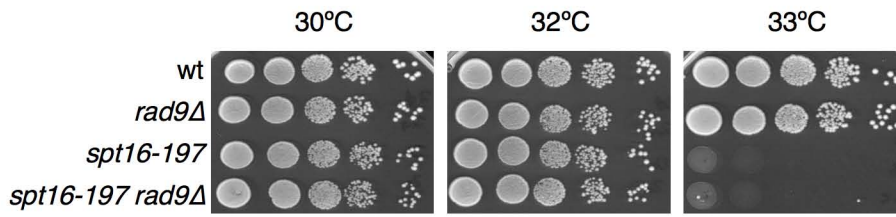

Figure S2

Supplement: Figure S2 — The G1 delay provoked by Spt16 inactivation was not prevented by the deletion of RAD9. (A) Wild-type (MMY20.4), rad9Δ (MMY20.1), spt16-197 (MMY20.2) and spt16-19 rad9Δ (MMY20.3) cells growing exponentially in YPD at 30°C were shifted to 37°C for four hours. Cells were then analyzed by flow cytometry. (B) Wild-type and mutant cells exponentially growing in YPD at 30°C were spotted onto YPD plates and incubated at 30°C, 32°C and 33°C, as indicated. (0.19 MB PDF) [file pgen.1000964.s002.pdf]

A

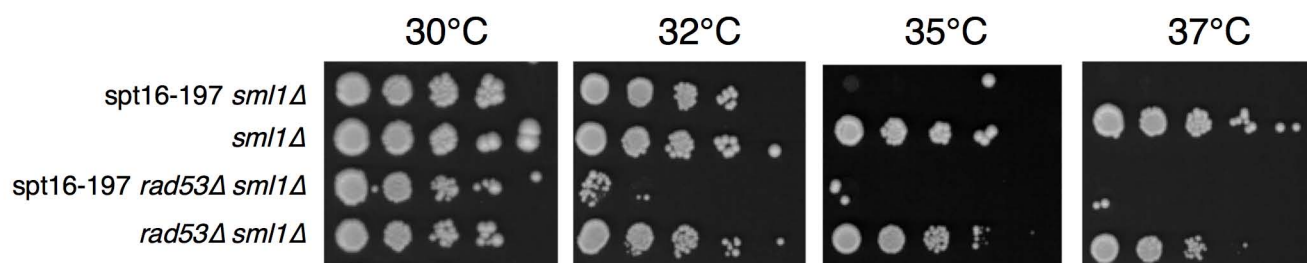

Figure S3

Supplement: Figure S3 — rad53Δ enhances the thermosensitivity of spt16-197 independently of the DNA damage checkpoint. Cells were grown in YPD medium at 25°C. 10-fold serial dilutions of the indicated strain were plated on YPD plates (or YPD+0.02% methyl methane sulfonate, MMS) and incubated for three days at the indicated temperatures. (0.17 MB PDF) [file pgen.1000964.s003.pdf]

A

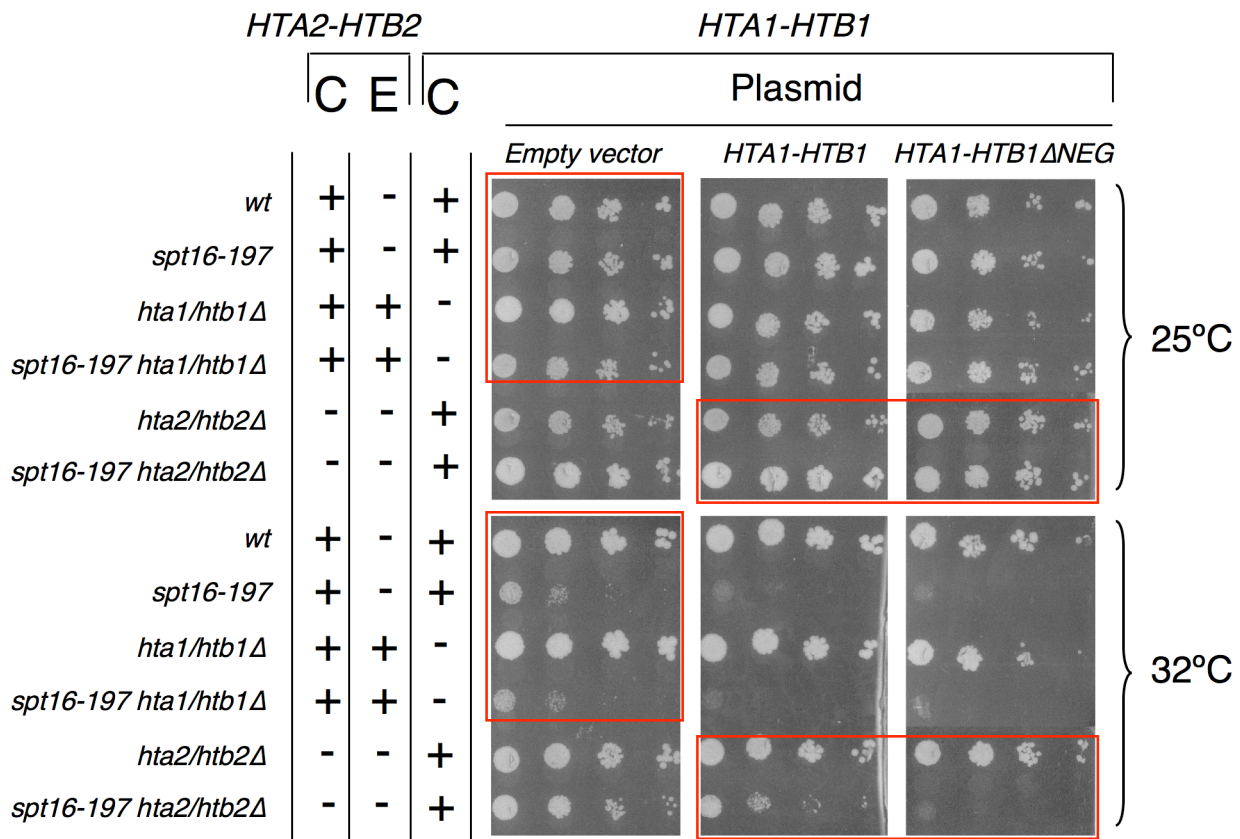

C —————> Chromosomal copy

E —————> Extrachromosomal copy of *HTA2-HTB2* (only in *hta1-htb1*Δ mutants)

B

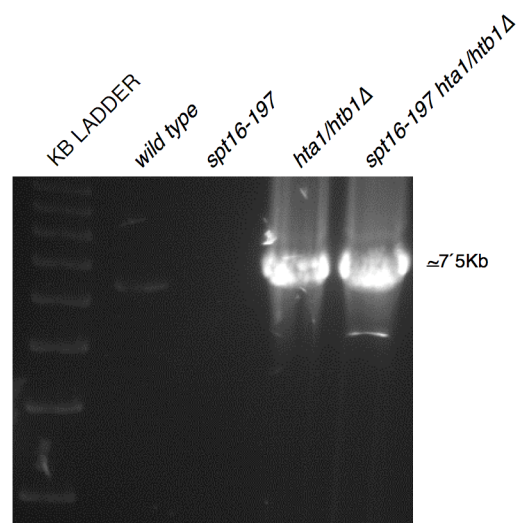

Figure S4

Supplement: Figure S4 — hta2/htb2Δ partially suppresses the thermosensitivity of spt16-197. (A) Strains FY120, FY348, FY710, DMY10, DMY11, and DMY12 were transformed with pRS316 (empty vector), pRS316-HTA1-HTB1 or pRS316-HTA1-HTB1ΔNEG. Transformants were grown in SC-Ura medium at 25°C. 10-fold serial dilutions of the indicated strain were plated on SC-Ura plates and incubated for three days at the indicated temperatures. The chromosomal, extrachromosal or plasmidic copies of HTA1–HTB1 and HTA2–HTB2 presented in each transformant are indicated. Red squares indicate the results of those strains containing two copies of H2A/H2B-encoding loci. (B) An extrachromosomal amplification of the HTA2–HTB2 locus was detected by PCR in FY710 (hta1/htb1Δ) and DMY11 (spt16-197 hta1/htb1Δ), following the protocol described in (Libuda and Winston, 2006). (1.22 MB PDF) [file pgen.1000964.s004.pdf]

A

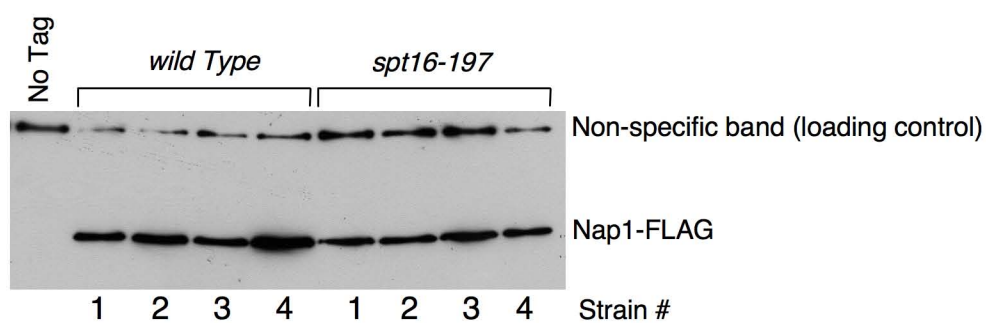

B

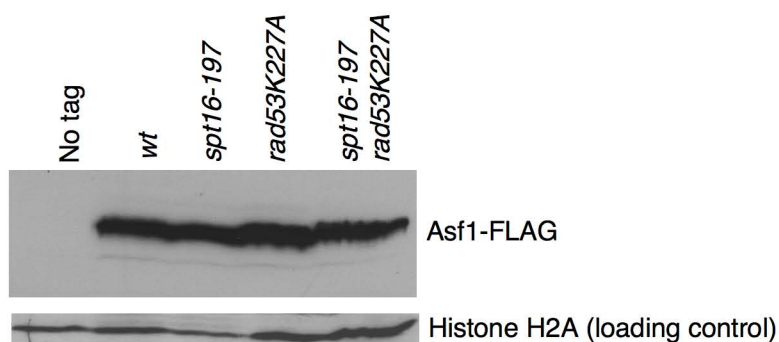

C

*ACT1* transcript levels normalized to rRNA in *spt16-197* Asf1-FLAG3 strain

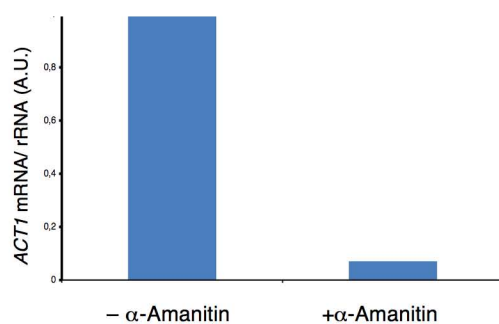

D

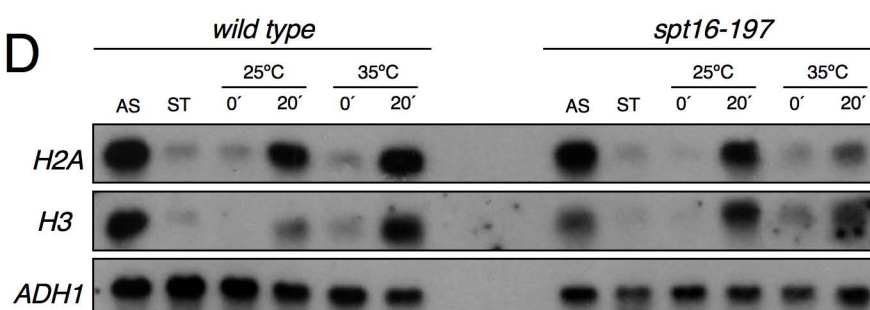

Figure S5

Supplement: Figure S5 — spt16-197 does not affect the levels of histone chaperones Nap1 and Asf1 or alter histone gene regulation. (A) Nap1 levels were unaffected in spt16-197. Nap1-FLAG was detected in WCEs of 4 independent generated wild type and spt16-197 strains by Western blotting using an anti-FLAG antibody. A non-specific band was used as the loading control. (B) Asf1 levels were unaffected in spt16-197. Asf1-FLAG was detected in WCE by Western blot with an anti-FLAG antibody. H2A was used as the loading control. (C) Alpha–amanitin inhibited RNA pol II transcription in alpha-factor-synchronized spt16-197 cells. The mRNA levels of the constitutively expressed ACT1 gene were measured by quantitative RT-PCR in the FY348 cells grown exponentially at 25°C and synchronized with alpha factor for four hours. The data was normalized to the levels of ribosomal RNA which is not affected by the concentration of alpha–amanitin used. (D) Repression of histones genes in G1 was not abolished by spt16-197. Wild-type (FY120) and spt16-197 (FY348) cells grown asynchronously (AS) were synchronized at START (ST) by treatment with alpha-factor for two hours at 25°C (ST), followed by an additional one hour at 25°C or 35°C in the presence of the mating pheromone. Cells were then released from the arrest at time 0 at either 25°C or 35°C by washing out the alpha-factor. Samples were taken at the indicated time points to analyze mRNA levels by Northern blot. H2A indicate the signal corresponding to HTA1 and HTA2, and H3 indicate the signal of HHT1 and HHT2. (0.24 MB PDF) [file pgen.1000964.s005.pdf]

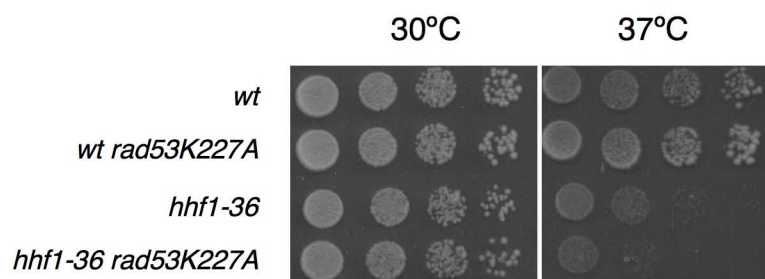

Figure S6

Supplement: Figure S6 — hhf1-36 and rad53K227A exhibit a negative synthetic interaction. Strains MSY623, DMY15, MSY781, and DMY16 exponentially grown in YPD at 30°C were spotted onto YPD plates and incubated for three days at 30°C and 37°C, as indicated. (0.16 MB PDF) [file pgen.1000964.s006.pdf]
