# Supplementary material for: FACT Prevents the Accumulation of Free Histones Evicted from Transcribed Chromatin and a Subsequent Cell Cycle Delay in G1
Source: PLoS Genet. 2010 May 20;6(5):e1000964. doi: 10.1371/journal.pgen.1000964 (PMC2873916; doi:10.1371/journal.pgen.1000964)
Supplement: Table S1 — The yeast strains used in this work. (0.03 MB DOC) [file pgen.1000964.s007.doc]

Table S1. The yeast strains used in this work.

| Strain | Genotype | Source |
| --- | --- | --- |
| BY4741 | *MATa his3∆1 leu2∆0 met15∆0 ura3∆0* | EUROSCARF |
| DMY1.1 | *MATa leu2-3/112 ura3-1 trp1-1 his3-11/15 ade2-1 can1-100 TRP1-GAL1,10-HHF1-FLAG-HHT1* | This study |
| DMY5 | FY2180 *sml1::NAT* | This study |
| DMY6 | FY2180 *rad53K227A* | This study |
| DMY7 | FY2180 *sml1::NAT rad53K227A* | This study |
| DMY8 | FY2180 *sml1::NAT mec1::KanMX4* | This study |
| DMY10 | FY120 *hta2-htb2::KanMX4* | This study |
| DMY11 | FY348 *hta1-htb1::LEU2* | This study |
| DMY12 | FY348 *hta2-htb2::KanMX4* | This study |
| DMY15 | MSY623 *rad53K227A* | This study |
| DMY16 | MSY781 *rad53K227A* | This study |
| FY120 | *MATa leu2∆1 ura3 his4-912∂ lys2-128∂* | (Hartzog et al. 1998) |
| FY2180 | *MATa leu2∆1 his4-912∂ lys2-128∂ FLAG-spt6-1004* | (Kaplan et al. 2003) |
| FY348 | *MATa leu2∆1 ura3 his4-912∂ lys2-128∂ spt16-197* | (Malone et al. 1991) |
| FY710 | *MATa, ura3-52 his4-912∂ lys2d-128 leu2∆1 hta1-htb1::LEU2* | (Malone et al. 1991) |
| FY98 | *MATa leu2∆1 ura3-52* | (Madison and Winston 1997) |
| MMY18.10 | *MATa leu2∆0 ura3∆0 his3∆0 lys2∂* | This study |
| MMY18.11 | *MATa leu2∆0 ura3∆0 lys2∆0 spt16-197* | This study |
| MMY18.12 | *MATa leu2∆0 ura3∆0 his3∆0 lys2∂ sic1::KanMX4* | This study |
| MMY18.9 | *MATa leu2∆0 ura3∆0 lys2∆0 spt16-197 sic1::KanMX4* | This study |
| MMY20.1 | *MATa leu2∆0 ura3∆0 lys2∆0 rad9::KanMX4* | This study |
| MMY20.2 | MATa leu2∆0 ura3∆0 spt16-197 | This study |
| MMY20.3 | *MATa leu2∆0 ura3∆0 his3∆0 lys2∆0 spt16-197 rad9::KanMX4* | This study |
| MMY20.4 | *MATa leu2∆0 ura3∆0 his3∆0 lys2∂* | This study |
| MSY623 | *MATa ∆(HHT1 HHF1) ∆(HHT2 HHF2) LEU2::(HHT1 HHF1)* *lys2∆201* *ura3-52* | (Santisteban et al, 1997) |
| MSY781 | *MATa ∆(HHT1 HHF1) ∆(HHT2 HHF2) LEU2::(HHT1 hhf1-36)* *lys2∆201* *ura3-52* | (Santisteban et al, 1997) |
| VO1 | FY120 *sml1::NAT* | This study |
| VO2 | FY120 *sml1::NAT mec1::KanMX4* | This study |
| VO3 | FY120 *rad53K227A* | This study |
| VO4 | FY120 *sml1::NAT rad53K227A* | This study |
| VO5 | FY348 *sml1::NAT* | This study |
| VO6 | FY348 *sml1::NAT mec1::KanMX4* | This study |
| VO7 | FY348 *rad53K227A* | This study |
| VO8 | FY348 *sml1::NAT rad53K227A* | This study |
| Y12690 | *MATa his3∆1 leu2∆0 lys2∆0 ura3∆0 sic1::KanMX4* | EUROSCARF |
| Y13576 | *MATa his3∆1 leu2∆0 lys2∆0 ura3∆0 rad9::KanMX4* | EUROSCARF |
