# Supplementary material for: FACT Prevents the Accumulation of Free Histones Evicted from Transcribed Chromatin and a Subsequent Cell Cycle Delay in G1
Source: PLoS Genet. 2010 May 20;6(5):e1000964. doi: 10.1371/journal.pgen.1000964 (PMC2873916; doi:10.1371/journal.pgen.1000964)
Supplement: Text S1 — Supplementary material and methods and supplementary references. (0.03 MB DOC) [file pgen.1000964.s008.doc]

##### Text S1

##### Supplementary Materials and Methods

**Yeast strains and general procedures.**

All the MMY strains were constructed by standard genetic tetrad analysis methods (Rose 1990). Strains MMY18 and MMY20 were generated by crossing FY348 with Y12690, and FY348 with Y13576, respectively. DMY 2 and DMY 3 were generated by crossing FY348 with 7982-5-1-K and FY710, respectively. The null allele *sml1∆::NAT* was constructed by integrating a PCR product containing nourseothricin (nat). The *rad53K227A* mutant was constructed by transforming the recipient strain with pCH3 linearized with *Eco*RI (Pellicioli et al. 1999). The *mec1∆::KAN* null mutation was constructed by co-transforming pKA-*MEC1* and pAN-*MEC1* digested by *Not*I (Schramke et al. 2001).

Cells were grown in yeast extract-peptone medium or in a synthetic complete medium with 2% glucose (2% raffinose or 2% galactose were used instead of glucose wherever indicated) (Rose 1990). To synchronize the cultures at START, mid log-phase cells were alpha-factor-treated for two hours (two additions of pheromone at 1µM separated by one hour). Wherever indicated, longer treatments or more additions, separated by one hour, were needed to synchronize the cultures at START.

A flow cytometry analysis of cells was performed as described using propidium iodide as a DNA stain (Winey et al. 1991). Stained cells were analyzed with a Becton Dickinson FACScan flow cytometer using the CELL QUEST software packages to collect and analyze the data (BDIS, San José, CA).

**Plasmids.** *p416ADH1-lacZ,* Ycplac33 and pRS316-Flag-yNap1 were described elsewhere (Gietz and Sugino 1988; Mumberg et al. 1995, Miyaji-Yamaguchi et al. 2003). *pTetoff-CLN3::HA* was constructed by cloning a HindIII-BamHI fragment from pCM187 (Gari et al. 1997) containing the Tet transactivator (tTA) at the unique SacI restriction site of plasmid pCM166, bearing a TEToff promoter-*CLN3* fusion (Gallego et al. 1997). The transcriptional fusions of *lacZ,* with different fragments of the intergenic region between *CYC3* and *CLN3* (plasmids pRS416-CLN3pr(1)::lacZ, pRS416-CLN3pr(2)::lacZ and pRS416-CLN3pr(3)::lacZ), were constructed by cloning the XbaI-KpnI fragment of p416GAL1-lacZ (Mumberg et al. 1994) containing the *lacZ* gene and the *CYC1* terminator at the same restriction sites in pRS416 (Sikorski and Hieter 1989). The different fragments of the *CLN3* promoter to be cloned were amplified by PCR by introducing a SacII restriction site at the 5´end and an XbaI restriction site at the 3´end. These fragments were inserted into the previous vector upstream from *lacZ* using the introduced restriction sites. Copy number stability was monitored by Q-PCR using an amplicon of the Amp gene of pR4316 (from 3391 to 3491 according to the published sequence of this plasmid (Sikorski and Hieter 1989)), in relation to the 5’ amplicon of the *GAL1* gene described in (Jimeno-González et al, 2006).

pRS316-HTA1-HTB1 and pRS316-HTA1-HTB1∆NEG were constructed by inserting the PCR-amplified *HTA1-HTB1* from wild-type or ∆NEG strains (Osley et al, 1986) into pRS316.

**References**

Formosa T, Ruone S, Adams MD, Olsen AE, Eriksson P et al. (2002) Defects in SPT16 or POB3 (yFACT) in Saccharomyces cerevisiae cause dependence on the Hir/Hpc pathway: polymerase passage may degrade chromatin structure. Genetics 162(4): 1557-1571.

Gallego C, Gari E, Colomina N, Herrero E, Aldea M (1997) The Cln3 cyclin is down-regulated by translational repression and degradation during the G1 arrest caused by nitrogen deprivation in budding yeast. Embo J 16(23): 7196-7206.

Gari E, Piedrafita L, Aldea M, Herrero E (1997) A set of vectors with a tetracycline-regulatable promoter system for modulated gene expression in Saccharomyces cerevisiae. Yeast 13(9): 837-848.

Gietz RD, Sugino A (1988) New yeast-Escherichia coli shuttle vectors constructed with in vitro mutagenized yeast genes lacking six-base pair restriction sites. Gene 74(2): 527-534.

Hartzog GA, Wada T, Handa H, Winston F (1998) Evidence that Spt4, Spt5, and Spt6 control transcription elongation by RNA polymerase II in Saccharomyces cerevisiae. Genes Dev 12(3): 357-369.

Jimeno-Gonzalez S, Gomez-Herreros F, Alepuz PM, Chávez S (2006) A gene-specific requirement for FACT during transcription is related to the chromatin organization of the transcribed region. Mol Cell Biol 26: 8710-8721.

Kaplan CD, Laprade L, Winston F (2003) Transcription elongation factors repress transcription initiation from cryptic sites. Science 301(5636): 1096-1099.

Libuda DE, Winston F (2006) Amplification of histone genes by circular chromosome formation in Saccharomyces cerevisiae. Nature 443: 1003-1007.

Madison JM, Winston F (1997) Evidence that Spt3 functionally interacts with Mot1, TFIIA, and TATA-binding protein to confer promoter-specific transcriptional control in Saccharomyces cerevisiae. Mol Cell Biol 17(1): 287-295.

Malone EA, Clark CD, Chiang A, Winston F (1991) Mutations in SPT16/CDC68 suppress cis- and trans-acting mutations that affect promoter function in Saccharomyces cerevisiae. Mol Cell Biol 11(11): 5710-5717.

Miyaji-Yamaguchi M, Kato K, Nakano R, Akashi T, Kikuchi A, Nagata K (2003) **Involvement of nucleocytoplasmic shuttling of yeast Nap1 in mitotic progression.** Mol Cell Biol 23(18): 6672-6684.

Mumberg D, Muller R, Funk M (1994) Regulatable promoters of Saccharomyces cerevisiae: comparison of transcriptional activity and their use for heterologous expression. Nucleic Acids Res 22(25): 5767-5768.

Mumberg D, Muller R, Funk M (1995) Yeast vectors for the controlled expression of heterologous proteins in different genetic backgrounds. Gene 156(1): 119-122.

Osley MA, Gould J, Kim S, Kane MY, Hereford L (1986). Identification of sequences in a yeast histone promoter involved in periodic transcription. Cell 45(4): 537-544.

Pellicioli A, Lucca C, Liberi G, Marini F, Lopes M et al. (1999) Activation of Rad53 kinase in response to DNA damage and its effect in modulating phosphorylation of the lagging strand DNA polymerase. Embo J 18(22): 6561-6572.

Rose MD, Winston, F. and Hieter, P. (1990) Methods in Yeast Genetics: A Laboratory Course Manual. Cold Spring Harbor, NY: Cold Sprong Harbor Laboratory Press.

Schramke V, Neecke H, Brevet V, Corda Y, Lucchini G et al. (2001) The set1Delta mutation unveils a novel signaling pathway relayed by the Rad53-dependent hyperphosphorylation of replication protein A that leads to transcriptional activation of repair genes. Genes Dev 15(14): 1845-1858.

Sikorski RS, Hieter P (1989) A system of shuttle vectors and yeast host strains designed for efficient manipulation of DNA in Saccharomyces cerevisiae. Genetics 122(1): 19-27.

Winey M, Goetsch L, Baum P, Byers B (1991) MPS1 and MPS2: novel yeast genes defining distinct steps of spindle pole body duplication. J Cell Biol 114(4): 745-754.
